# Targeting TNF/TNFR superfamilies in immune-mediated inflammatory diseases

**DOI:** 10.1084/jem.20240806

**Published:** 2024-09-19

**Authors:** Praveen Krishna Veerasubramanian, Thomas A. Wynn, Jie Quan, Fridrik J. Karlsson

**Affiliations:** 1 Inflammation and Immunology Research Unit, Pfizer, Inc., Cambridge, MA, USA

## Abstract

Dysregulated signaling from TNF and TNFR proteins is implicated in several immune-mediated inflammatory diseases (IMIDs). This review centers around seven IMIDs (rheumatoid arthritis, systemic lupus erythematosus, Crohn’s disease, ulcerative colitis, psoriasis, atopic dermatitis, and asthma) with substantial unmet medical needs and sheds light on the signaling mechanisms, disease relevance, and evolving drug development activities for five TNF/TNFR signaling axes that garner substantial drug development interest in these focus conditions. The review also explores the current landscape of therapeutics, emphasizing the limitations of the approved biologics, and the opportunities presented by small-molecule inhibitors and combination antagonists of TNF/TNFR signaling.

## Teaser

Following over two decades of clinical success for anti-TNF drugs, antagonists against multiple TNF/TNFR superfamily proteins are in development for IMIDs. This review explores the promise of these signaling molecules and highlights the opportunities and challenges in this fast-evolving area.

## Introduction

Immune-mediated inflammatory diseases (IMIDs) are a collection of disorders characterized by an exaggerated and dysregulated immune response that causes chronic inflammation and tissue damage. These debilitating ailments significantly compromise the quality of life and cause substantial morbidity (Kwon et al., 2023). Recent scientific endeavors have diligently focused on elucidating the intricate molecular pathways underlying these pathological states and addressing them with novel therapeutics. Notably, the tumor necrosis factor (TNF) and tumor necrosis factor receptor (TNFR) superfamilies (summarized in Table S1), comprising 18 ligands and 29 receptors, respectively, play pivotal roles as mediators of proinflammatory and apoptotic signaling within diverse immune cell populations (van Loo and Bertrand, 2023).

**Figure 1.**
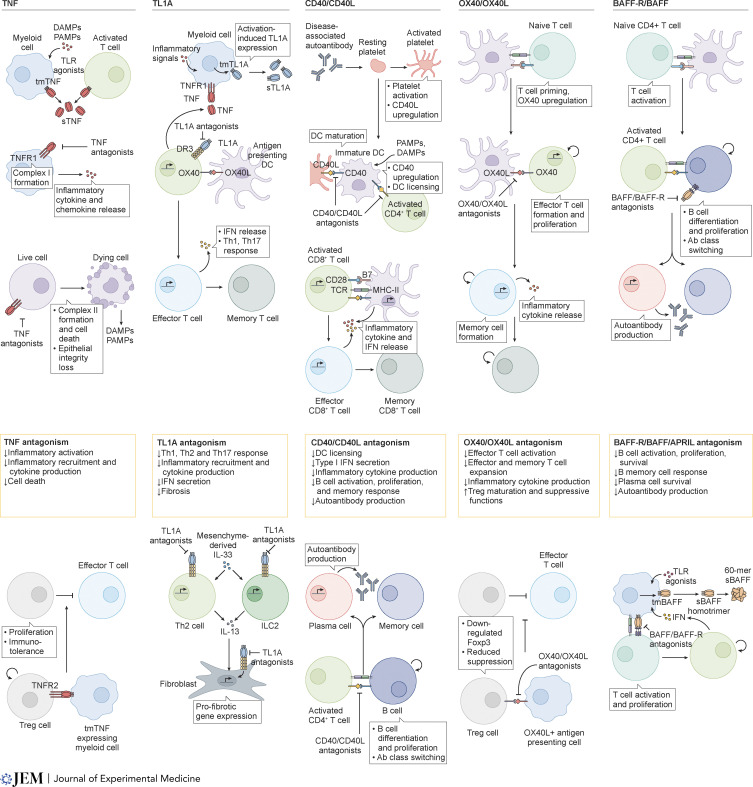
**Molecular signaling mechanisms of the focus TNF/TNFR molecules and the anti-inflammatory benefits of their antagonism.** TNF: TNF is produced mainly by DAMP/PAMP-activated myeloid cells. Myeloid cells respond to soluble TNF/TNFR1 signaling by recruiting the proinflammatory complex I, orchestrating the release of inflammatory cytokines. Under conditions of loss of cell death checkpoints, TNF/TNFR1 signaling causes the formation of the cytotoxic complex II that precipitates cellular death, fueling chronic inflammation. TNFR2 agonism on Tregs stimulates their proliferation, resulting in immunotolerance via suppression of effector T cells. TL1A: Myeloid activation through inflammatory signals such as TNF induces TL1A expression. This drives T cell effector function, memory cell formation, and IFN production through a Th1/Th17 response. Along with IL-33, TL1A catalyzes the ILC2-mediated Th2 response, and the consequent IL-13 production triggers matrix metalloproteinase production, tissue remodeling, and fibrosis. CD40/CD40L: CD40 signaling results in DC maturation and licensing, causing them to secrete inflammatory cytokines and type I IFN directly and indirectly through CD8^+^ T cell activation and proliferation. CD40 signaling in B cells drives B cell proliferation and differentiation into memory cells and plasma cells that are responsible for autoantibody production. OX40/OX40L: OX40 signaling in T cells drive effector and memory T cell formation and inflammatory cytokine production. In Tregs, OX40 signaling downregulates Foxp3 expression, impairing Treg-mediated effector T cell suppression. BAFF/BAFF-R: Activated myeloid cells produce BAFF that oligomerizes and induces T cell activation and proliferation. BAFF also plays a leading role in costimulating B cells to drive proliferation, differentiation, and autoantibody production. MHC II, major histocompatibility complex II; PAMP, pathogen-associated molecular patterns; TGF-β, transforming growth factor-β.

Several TNF superfamily ligands and receptors are upregulated in immune cells in the presence of proinflammatory cues. TNF superfamily ligands binding to their respective receptors on target cells initiate intracellular signaling cascades that activate pivotal transcription factors, including nuclear factor-κB (NF-κB) and activator protein (AP-1) (Shi and Sun, 2018). This orchestrates transcription that facilitates inflammation and immune effector functions (Fig. 1). TNF family receptors signal through intracellular recruitment of mediator complexes through their tumor necrosis factor receptor–associated factor (TRAF) binding motif, or death domain. Dysregulated TNF/TNFR signaling has been implicated in the progression of IMIDs, driving perpetual inflammation and consequential tissue damage. Furthermore, TNF/TNFR members play crucial roles in regulating T cell responses by providing costimulatory and coinhibitory signals originating from antigen-presenting cells (APCs) (So and Ishii, 2019). Signaling from the various TNF/TNFR interactions facilitates immune homeostasis and attenuates autoimmune responses by modulating the intensity and duration of T cell responses. T cell activation mediated by costimulatory signals promotes their expansion, survival, and differentiation into memory cells. Certain TNF/TNFR interactions may also induce reverse signaling within APCs and B cells, resulting in their enhanced activation, proliferation, and maturation (So and Ishii, 2019). Accordingly, therapeutic interventions aimed at antagonizing TNF/TNFR signaling have gained considerable attention in managing several inflammatory conditions. Of note are biologics that help manage disease symptoms and improve patient prognosis.

Despite substantial strides in comprehending the roles of TNF/TNFR members in IMIDs, numerous aspects of their precise molecular mechanisms remain to be elucidated. Understanding the intricate interplay between TNF family ligands, their cognate receptors, and downstream signaling pathways is crucial for developing novel therapeutic strategies to combat IMIDs. In this review, we outline the roles of five pivotal TNF/TNFR targets, namely TNF, TL1A, CD40/CD40L, OX40/OX40L, and BAFF-R/BAFF axes. These proteins have been intensely investigated in preclinical and clinical studies as therapeutic targets for a variety of IMIDs, including atopic dermatitis (AD), asthma, inflammatory bowel diseases (IBD) such as Crohn’s disease (CD) and ulcerative colitis (UC), psoriasis, systemic lupus erythematosus (SLE), and rheumatoid arthritis (RA) (Table 1). This assortment of chronic inflammatory diseases manifests with multifactorial pathogenesis arising from genetic, environmental, and immunological influences and forms the bulk of the focus of this review. Multiple TNF/TNFR members are significantly upregulated in IMID diseases. In parallel, genome-wide association studies (GWAS) also shed light on the involvement of several TNF/TNFR genes in our focus IMIDs (Table 2). Taken together, these data reveal the extensive involvement of TNF and TNFR superfamilies in inflammatory diseases.

**Table 1.** Drugs in various stages of development targeting TNF/TNFR for the focus IMIDs (phase 2 and above)

Drug (Trade name)	Target and action	Form	Stage and indications
TNF axis			
ABBV-3373	TNF antagonism, glucocorticoid receptor modulation	Adalimumab-GRM steroid conjugate (ADC)	Phase 2: RA
Adalimumab (Humira)	TNF antagonism	Human IgG1 κ mAb	Launched: CD, psoriasis, RA, UC
Certolizumab pegol (Cimzia)	TNF antagonism	PEGylated anti-TNF F(ab) fragment of humanized IgG1 κ mAb	Launched: CD, psoriasis, RA; Phase 2: UC
Etanercept (Enbrel)	TNF antagonism	Fusion of extracellular domain of human TNFR2 and the Fc portion of human IgG1 Ab	Launched: psoriasis, RA
Golimumab (Simponi)	TNF antagonism	Human IgG1 κ mAb	Launched: UC, RA; Phase 2: CD
Infliximab (Remicade)	TNF antagonism	Humanized IgG1 κ mAb	Launched: CD, psoriasis, RA, UC
Ozoralizumab (Nanozora)	TNF antagonism	Humanized trivalent, bispecific nanobody made of two anti–TNFα V_H_Hs and an anti–HSA V_H_H	Launched: RA (Japan)
SAR441566	TNF antagonism	Small molecule	Phase 2: RA, psoriasis
TL1A axis			
RG6631	TL1A antagonism	Human IgG1 mAb	Phase 2: UC, CD
TEV-48574	TL1A antagonism	Human IgG1 mAb	Phase 2: UC, CD
Tulisokibart	TL1A antagonism	Humanized IgG1 mAb	Phase 2: UC, CD
CD40/CD40L axis			
Abiprubart	CD40 antagonism	Humanized Fc-silenced IgG4 mAb	Phase 2: RA
Dapirolizumab pegol	CD40L antagonism	PEGylated anti-CD40L F(ab’) fragment of humanized mAb	Phase 3: SLE
Dazodalibep	CD40L antagonism	Fusion of two tenascin-3 Fnlll domains (Tn3) with human serum albumin	Phase 2: RA
Frexalimab	CD40L antagonism	Humanized mAb	Phase 2: SLE
Iscalimab	CD40 antagonism	Human Fc-silenced IgG1 mAb	Phase 2: SLE
Ravagalimab	CD40 antagonism	Humanized IgG1 mAb	Phase 2: UC
OX40/OX40L axis			
Amlitelimab	OX40L antagonism	Human IgG4 mAb	Phase 3: AD, Phase 2: Asthma
IMG-007	OX40 antagonism	Humanized Fc modulated IgG1 mAb	Phase 1/2: AD
Rocatinlimab	OX40 antagonism	Human IgG1 mAb	Phase 3: AD
Telazorlimab	OX40 antagonism	Humanized IgG1 mAb	Phase 2: AD
BAFF/BAFF-R axis			
Belimumab (Benlysta)	BAFF antagonism	Human IgG1 λ mAb	Launched: SLE
Ianalumab	BAFF-R antagonism	Human IgG1 κ mAb	Phase 3: SLE
Povetacicept	BAFF and APRIL neutralization	Fusion of engineered TACI receptor and the Fc portion of human IgG Ab	Phase 2: SLE
Rozibafusp alfa	BAFF and ICOSL antagonism	Bispecific IgG2 Ab-peptide conjugate that targets BAFF and ICOSL	Phase 2: SLE
Telitacicept	BAFF and APRIL neutralization	Fusion of the TACI receptor and the Fc portion of human IgG Ab	Phase 3: RA, SLE. Launched: SLE (China)

ICOSL, inducible T cell costimulator ligand; V_H_H, variable region of heavy-chain-only antibody.

**Table 2.** Representative GWAS associations for TNF/TNFR superfamilies’ genes in focus inflammatory diseases

Variant	P value	Mapped TNF gene(s)	Disease trait(s)	Study
Ligands				
rs755023315	3 × 10^−9^	*CD70*	Asthma	Han et al. (2020)
rs78037977	6 × 10^−13^	*FASLG*	Asthma (child onset)	Ferreira et al. (2019)
rs12118303	3 × 10^−10^	*FASLG*	Psoriasis	Tsoi et al. (2017)
rs12068671	7 × 10^−8^	*FASLG*	SLE	Langefeld et al. (2017)
rs2844482	1 × 10^−9^	*LTA*	Asthma	Zhu et al. (2019)
rs11811788	2 × 10^−39^	*TNFSF4*	AD	Budu-Aggrey et al. (2023)
rs10158467	8 × 10^−29^	*TNFSF4*	Asthma (child onset)	Ferreira et al. (2019)
rs6681482	4 × 10^−12^	*TNFSF4*	RA	Ishigaki et al. (2022)
rs2205960	3 × 10^−90^	*TNFSF4*	SLE	Yin et al. (2021)
rs7848647	1 × 10^−25^	*TNFSF15*	Psoriasis, UC, CD, ankylosing spondylitis, sclerosing cholangitis	Ellinghaus et al. (2016)
rs34187268	3 × 10^−9^	*TNFSF15*	Asthma	Han et al. (2020)
rs4246905	1 × 10^−8^	*TNFSF15*	SLE, psoriasis, UC, CD, juvenile idiopathic arthritis, type 1 diabetes mellitus	Li et al. (2015)
rs6478106	5 × 10^−46^	*TNFSF15*	CD	Yamazaki et al. (2013)
rs10817678	6 × 10^−88^	*TNFSF15*	CD, leprosy	Jung et al. (2022)
rs2006996	4 × 10^−13^	*TNFSF15*	UC, CD	Okada et al. (2011)
rs9286879	6 × 10^−22^	*TNFSF18*	CD	Jostins et al. (2012)
rs1799964	1 × 10^−11^	*TNF*, *LTA*	Asthma (childhood onset)	Zhu et al. (2020)
rs1799964	4 × 10^−11^	*TNF*, *LTA*	CD	Franke et al. (2010)
rs1800630	3 × 10^−14^	*TNF*, *LTA*	RA, COVID-19	Yao et al. (2023)
Receptors				
rs1883832	4 × 10^−12^	*CD40*	Psoriasis, UC, CD, ankylosing spondylitis, sclerosing cholangitis	Ellinghaus et al. (2016)
rs6074022	3 × 10^−12^	*CD40*	CD	Liu et al. (2015)
rs1883832	4 × 10^−21^	*CD40*	RA	Ishigaki et al. (2022)
rs4810485	1 × 10^−8^	*CD40*	SLE	Langefeld et al. (2017)
rs11616188	3 × 10^−14^	*LTBR*	Psoriasis, UC, CD, ankylosing spondylitis, sclerosing cholangitis	Ellinghaus et al. (2016)
rs7954567	5 × 10^−9^	*LTBR*	CD	de Lange et al. (2017)
rs1860545	3 × 10^−17^	*TNFRSF1A*	Psoriasis, UC, CD, ankylosing spondylitis, sclerosing cholangitis	Ellinghaus et al. (2016)
rs6010620	5 × 10^−18^	*TNFRSF6B*	AD	Sakaue et al. (2021)
rs6062496	2 × 10^−30^	*TNFRSF6B*	Psoriasis, UC, CD, ankylosing spondylitis, sclerosing cholangitis	Ellinghaus et al. (2016)
rs6011033	1 × 10^−12^	*TNFRSF6B*	Asthma	Han et al. (2020)
rs2230624	8 × 10^−13^	*TNFRSF8*	Asthma	Olafsdottir et al. (2020)
rs1201113	7 × 10^−11^	*TNFRSF8*	Eczema	Kichaev et al. (2019)
rs227163	3 × 10^−9^	*TNFRSF9*	RA	Okada et al. (2014)
rs7014637	2 × 10^−8^	*TNFRSF10D*	Asthma	Chang et al. (2023)
rs4574025	7 × 10^−10^	*TNFRSF11A*	AD	Budu-Aggrey et al. (2023)
rs4574025	2 × 10^−10^	*TNFRSF11A*	Asthma	Pividori et al. (2019)
rs8086340	3 × 10^−10^	*TNFRSF11A*	Eczema	Kichaev et al. (2019)
rs371734407	4 × 10^−8^	*TNFRSF11A*	RA	Ishigaki et al. (2022)
rs35966917	5 × 10^−9^	*TNFRSF13B*	SLE	Yin et al. (2021)
rs2234161	2 × 10^−11^	*TNFRSF14*	Psoriasis, UC, CD, ankylosing spondylitis, sclerosing cholangitis	Ellinghaus et al. (2016)
rs10910095	4 × 10^−13^	*TNFRSF14*	Asthma, eczema, allergic rhinitis	Johansson et al. (2019)
rs2258734	2 × 10^−24^	*TNFRSF14*	RA	Ishigaki et al. (2022)
rs10910092	1 × 10^−11^	*TNFRSF14*	UC	Liu et al. (2015)

Data from GWAS Catalog (Sollis et al., 2023).

In this review, we will elucidate the causes and downstream consequences of activation of these five TNF/TNFR molecular axes of interest, their roles in specific immune cell subsets, contributions to the focus IMIDs, and the significant therapeutic development efforts against them. We will also sum up the opportunities and challenges existing in the therapeutic landscape involving the use of TNF/TNFR-targeted drugs in inflammatory diseases.

## Promising TNF/TNFR targets in inflammatory diseases

### TNF

#### Signaling mechanism

Originally described in 1975 when scientists discovered it as a macrophage-secreted factor inducing necrotic cell death in certain tumors, TNF has since been recognized as a cardinal proinflammatory cytokine with context-dependent effects, including inflammatory response, survival, and apoptosis in its target cells (Carswell et al., 1975). TNF is produced by macrophages, T cells, B cells, neutrophils, natural killer (NK) cells, endothelial cells, and fibroblasts under inflammatory conditions. Both membrane-bound TNF and its soluble form produced by TNF-α-converting enzyme (TACE) proteolysis can induce pathological inflammation in various chronic inflammatory and autoimmune conditions. TNF acts through its two receptors, tumor necrosis factor receptor 1 (TNFR1, encoded by *TNFRSF1A*) and tumor necrosis factor receptor 2 (TNFR2, encoded by *TNFRSF1B*). TNFR1 is expressed ubiquitously, while TNFR2 is restricted to regulatory T cells (Tregs), NK cells, CD4^+^ and CD8^+^ T cells, and endothelial cells (Naserian et al., 2020). Lymphotoxin alpha (LT-α, encoded by *LTA*), a paralog of TNF, is a cytokine released by activated lymphocytes that binds to TNFR1, TNFR2, and HVEM as a homotrimer, and to LTβR as a heterotrimer with lymphotoxin beta (LT-β, encoded by *LTB*). While some studies show LT-α to be less efficient than TNF in activating TNFR1 and inducing cell death and inflammatory transcription through NF-κB (Andrews et al., 1990; Chaturvedi et al., 1994), others show no differences in TNFR1 stimulation potency between TNF and LT-α (Etemadi et al., 2013). In inflammatory diseases, TNFR1 activation by soluble and membrane-bound forms of TNF induces cell death, immune cell activation, and the expression of cytokines (such as IL-1, IL-6), chemokines (such as IL-8, RANTES), matrix metalloproteinases (such as MMP9), and adhesion molecules (such as ICAM-1, E-selectin). TNFR1 can also indirectly contribute to inflammation by causing lytic cell death, such as apoptosis-driven necrosis, pyroptosis, and necroptosis, consequently releasing damage-associated molecular patterns (DAMPs), and activating pattern-recognition receptors in neighboring bystander cells (Annibaldi and Meier, 2018; van Loo and Bertrand, 2023). Notably, TNFR1 signaling-associated cell death disrupting the dermal or intestinal epithelial barrier may lead to microbial invasion, thereby perpetuating inflammation. Taken together, TNF/TNFR1 signaling can induce inflammation directly through inflammatory transcription and indirectly via cell death.

TNFR2 signaling triggered by membrane-bound TNF leads to TRAF-2 recruitment and non-canonical NF-κB and MAPK pathways initiation, culminating in the proliferation, survival, and differentiation of T and B lymphocytes. In CD4^+^ T helper and CD8^+^ T effector cells, TNF/TNFR2 interactions act as co-stimulatory signals mediating proliferation and effector functions including the production of TNF, IFN-γ, and IL-2 following T cell receptor (TCR) activation (Kim et al., 2006). In Tregs, TNFR2 stimulation is tied to IL-2 production and proliferation via activation of the NF-κB and MAPK pathways (He et al., 2018; Wang et al., 2018). Importantly, Treg expression of TNFR2 is among the highest in all cell types and correlates with immunotolerance and anti-inflammatory functions, suggesting that TNFR2 agonism and consequent Treg proliferation may be therapeutic in autoimmune and chronic inflammatory conditions (Faustman and Davis, 2010).

#### Disease relevance

Knockout studies have demonstrated that TNF plays pivotal roles in innate and humoral immune responses, including pathogen defense, inflammation resolution, and tissue repair, and is necessary for proper lymphoid-organ and germinal center development (Marino et al., 1997; Pasparakis et al., 1996). Chronic inflammation induced by aberrant expression of TNF is a pathological hallmark of several IMIDs. Elevated levels of TNF seen in the serum and diseased tissue of patients with inflammatory diseases, including RA and IBD, correlate with disease severity (Maeda et al., 1992; Robak et al., 1998). In addition, polymorphisms in *TNFRSF1A* are associated with psoriasis, UC, and CD (Table 2).

#### Drug development

TNF antagonistic biologics have been in development for three decades and are approved for inflammatory conditions, including CD, psoriasis, RA, and UC (Elliott et al., 1993). Adalimumab, a human anti-TNF monoclonal antibody (mAb), is approved for CD, psoriasis, RA, and UC. Certolizumab pegol is a pegylated anti-TNF F(ab’) fragment of a humanized mAb that has been launched for CD, psoriasis, and RA treatment. Golimumab is a human anti-TNF mAb that is approved for UC and RA treatments. Infliximab is a humanized anti-TNF mAb that is indicated for CD, psoriasis, RA, and UC. Whereas the other approved anti-TNF biologics are mAbs, etanercept is a fusion product of two extracellular domains of TNFR2 and the Fc region of human IgG1 Ab that is indicated for psoriasis and RA. Pivotal studies that led to the approval of these anti-TNF biologics are summarized in Table S2. Novel TNF antagonists that are currently being studied in the clinic are highlighted below.

Ozoralizumab (ATN-103) is a humanized trivalent, bispecific biologic comprising of two anti-TNF nanobody (single-domain antibody) fragments in addition to an anti-human serum albumin (HSA) nanobody that extends its half-life. In 2010, it underwent a phase 2 evaluation in 266 RA patients where it was found that subcutaneous monthly ozoralizumab was safe and induced clinical remission in 38% of patients (NCT01063803) (Fleischmann et al., 2012). After successful phase 3 trials in Japan, the drug has been approved for treating RA in Japan. In a study comprising 381 patients who were non-responders to methotrexate (JapicCTI-184029), 30 or 80 mg ozoralizumab was administered subcutaneously monthly and a clinical response was achieved in significantly more subjects (79.6% and 75.3% of subjects, P < 0.001) compared with placebo (37.3%) at week 16 (Takeuchi et al., 2022). Another open-label phase 3 trial in RA patients also established that both doses of ozoralizumab were safe and efficacious even without methotrexate administration, and clinical improvements were maintained through 52 wk (JapicCTI-184031) (Tanaka et al., 2023).

Antibody–drug conjugates (ADCs) are innovative therapeutics that permit targeted delivery of drug payloads. Systemic glucocorticoid treatments broadly used for inflammation management raise risks of serious adverse events (SAEs) that may be avoided by its selective delivery to inflamed tissues with elevated TNF levels. The first ADC being developed for IMIDs, ABBV-3373, is composed of adalimumab conjugated with a glucocorticoid receptor modulator (GRM) steroid. A recently completed phase 2a study in 47 active RA patients on methotrexate treatment evaluated the effects of either 100 mg intravenous ABBV-3373 or 80 mg subcutaneous adalimumab every 2 wk (NCT03823391). ABBV-3373 significantly reduced disease activity at week 12 when compared with historical adalimumab treatment (−2.65 versus −2.13, P = 0.022) but performed only marginally better than in-trial adalimumab treatment (−2.51, non-significant) (Buttgereit et al., 2023). ABBV-3373 was found to possess a similar safety profile to in-trial adalimumab (Buttgereit et al., 2023). However, no further development of ABBV-3373 has since been reported and the clinical development of a more stable derivative, ABBV-154, has ceased. ABBV-154 was in phase 2b trials for CD (NCT05068284) and RA (NCT04888585) when the trials were strategically terminated after it was determined through risk-benefit analyses that the ADC did not outperform adalimumab.

SAR441566 is a novel small molecule TNF inhibitor that stabilizes an asymmetric conformation of soluble TNF trimer causing it to bind to only two out of three possible TNFR1 receptor binding sites, consequently limiting receptor clustering and signaling (McMillan et al., 2021; Vugler et al., 2022). In a preclinical murine model of collagen-induced arthritis (CIA), SAR441566 exhibited dose-dependent suppression of disease symptoms. Oral doses of 10 and 30 mg/kg of SAR441566 elicited a reduction of 68% and 84%, respectively, in the disease score, compared with a placebo (Vugler et al., 2022). The drug became the first small molecule TNF inhibitor to enter clinical trials with the ongoing recruitment for phase 2 trials in an estimated 240 patients with RA (NCT06073093) and in an estimated 207 patients with plaque psoriasis (NCT06073119). Improvements in hit-finding techniques have led to the recent discovery of several small molecule inhibitors of TNF, as discussed in Opportunities and challenges in TNF/TNFR drug therapy for inflammatory diseases section.

Since its first approval over 25 years back, TNF inhibition has remained one of the most widely used and successful anti-inflammatory therapeutics to control RA, IBD, and psoriasis. Efforts focused on refining treatment strategies using approved TNF antagonists, as well as ongoing development of novel TNF therapeutics attest to the importance of TNF as a pre-eminent target in several IMIDs.

### TL1A

#### Signaling mechanism

TNF-like ligand 1A (TL1A, encoded by *TNFSF15*) is an inflammatory cytokine expressed primarily by APCs such as dendritic cells (DCs) and macrophages upon inflammatory stimulation through Toll-like receptor (TLR) ligands, IFN-γ, and FcγR activation (Cassatella et al., 2007). It is also expressed to a lesser degree by vascular endothelial cells (Al-Lamki et al., 2008). Membrane-bound TL1A undergoes proteolytic cleavage, ectodomain shedding, and solubilization in a process thought to be primarily TACE-dependent. TL1A signals through death receptor 3 (DR3, encoded by *TNFRSF25*) expressed on activated lymphocytes, NK cells, NKT cells, innate lymphoid cells (ILCs), fibroblasts, and epithelial cells (Tougaard et al., 2016). In resting T cells, the death domain (DD) of DR3 is suppressed by SODD (Jiang et al., 1999). In activated T cells, TL1A ligation triggers rapid NF-κB, PI3K, and c-Jun N-terminal kinase pathway-mediated inflammation via TNFR-associated DD signaling. Subsequently, a delayed-onset Fas-associated DD recruitment causes caspase signaling and apoptotic cell death (Wang et al., 2014). Interestingly, NF-κB activation may activate cIAP, a negative regulator of TL1A-mediated apoptosis, preferentially in lymphocytes (Xu et al., 2022). TL1A also has a decoy receptor, the soluble DcR3, which neutralizes TL1A and antagonizes its effects. Taken together, TL1A signaling plays a multifaceted role in inflammatory activation.

#### Disease relevance

TL1A-DR3 interaction acts as a potent costimulation signal in T cells, amplifying effector T cell pathways (Tougaard et al., 2016). TL1A induces the secretion of proinflammatory cytokines such as TNF, contributing to aggravated inflammation. TL1A signaling is associated with the pathogenesis of T-cell-mediated autoimmune diseases, such as RA, SLE, and psoriasis. Genetic variants of the *TNFSF15* gene are associated with psoriasis, UC, CD, SLE, asthma, and juvenile idiopathic arthritis (Table 2). Serum levels of soluble TL1A and intestinal levels of membrane-bound TL1A and DR3 are elevated in both UC and CD patients (Bamias et al., 2010, 2012; Prehn et al., 2004). Specifically, TL1A upregulation was seen in macrophages and lamina propria lymphocytes in colon tissue from CD patients and correlated to disease activity (Bamias et al., 2003). TL1A also enhanced IFN-γ secretion by phytohemagglutinin-activated lamina propria mononuclear cells isolated from lesional CD tissue in comparison with non-diseased and non-lesional diseased controls, suggesting a role for TL1A in maintaining IFN-γ–dependent Th1 responses seen in patient colons (Bamias et al., 2003). TL1A drives inflammation by inducing TNF production and synergizing with IL-12 and IL-18 to enhance IFN-γ production in T cells and NK cells (Papadakis et al., 2004). TL1A signaling is also believed to contribute to mucosal barrier disruption, as seen in its ability to increase the barrier permeability of TNF-treated Caco-2 cells and mucosa of dextran sulfate sodium–induced colitis mice by affecting tight junction proteins (Yang et al., 2019). While TL1A enhances Treg proliferation, it attenuates the ability of Treg to suppress conventional T cells (Taraban et al., 2011). TL1A has also been reported to induce fibrosis by inducing collagen synthesis in fibroblasts, potentially through TGF-β/Smad3 signaling (Jacob et al., 2020; Li et al., 2018). Together with mesenchyme-derived IL-33, TL1A induces IL-13 secretion by ILC type 2 (ILC2) and Th2 cells (Hassan-Zahraee et al., 2022). This catalyzes fibrosis and tissue remodeling by driving macrophage production of TGF-β and matrix metalloproteinase (MMP) (Hassan-Zahraee et al., 2022). The strong association of TL1A with IBD immunopathogenesis has paved the way for clinical studies assessing TL1A antagonism for UC and CD treatment.

#### Drug development

RG6631 (PF-06480605/RVT-3101) is a human IgG1 mAb antagonist for TL1A under investigation for UC and CD treatment. In a phase 2a study involving 50 UC patients (NCT02840721), intravenous administration of 500 mg RG6631 resulted in endoscopic improvement at week 14 in 38.2% of participants, a significantly better response than the historical placebo groups (6%, P < 0.001) based on two phase 3 induction studies of tofacitinib (Danese et al., 2021). The drug was well tolerated, with an acceptable short-term safety profile (Danese et al., 2021). Anti-TL1A therapy reduced free intestinal TL1A levels in both responders and non-responders and downregulated Th1, Th2, and IL-23 pathways in responders (Hassan-Zahraee et al., 2022). Additionally, fibrosis and extracellular remodeling genes were downregulated with RG6631 treatment, highlighting the relevance of TL1A signaling-induced fibrosis in UC (Hassan-Zahraee et al., 2022). A recently concluded 56-wk-long phase 2b evaluation of RG6631 involving 246 participants with UC demonstrated that the drug was well tolerated and showed a favorable safety profile at all tested dosages (NCT04090411). At week 14, 32% of the subjects achieved clinical remission compared with 12% in placebo group (P = 0.01), and 40% achieved endoscopic improvement compared with 19% in the placebo group (P = 0.01) in the pooled drug cohort (Roivant Sciences Ltd., 2023a). Clinical remission was maintained in the treatment group by week 56 of study, and no patient receiving the expected phase 3 dose developed neutralizing antibodies (Roivant Sciences Ltd., 2023b). RG6631 is also being investigated in a phase 2 trial involving 105 participants with CD (NCT05910528).

Tulisokibart (MK-7240/PRA-023) is a humanized IgG1 anti-TL1A mAb evaluated in UC and CD phase 2 studies. In a phase 2a non-placebo-controlled study involving 55 subjects with CD (NCT05013905), patients received 1,000 mg of the drug intravenously on day 1, followed by 500 mg at weeks 2, 6, and 10. Tulisokibart treatment resulted in 26% endoscopic response and 49% clinical remission (Feagan et al., 2023a). No SAEs were considered treatment related. In a phase 2 study in 135 UC patients (NCT04996797), 1,000 mg tulisokibart was administered intravenously on day 1, followed by 500 mg at weeks 2, 6, and 10. At week 12, drug treatment achieved significantly higher clinical remission and endoscopic improvement at 26.5% and 36.8%, respectively, compared with placebo group rates of 1.5% and 6%, respectively (Sands et al., 2023). Rates of treatment-emergent AEs were comparable between the drug and placebo treatments, indicating that the drug was well-tolerated. A phase 3 study involving 1020 UC subjects has been announced (NCT06052059).

TEV-48574 is a human IgG1 anti-TL1A mAb that recently started recruiting for phase 2 trials in UC and CD (NCT05668013 and NCT05499130). Notably, TEV-48574 was the subject of a phase 2 study in asthma that was terminated after failing to meet primary objectives at interim analysis (NCT04545385).

In summary, TL1A antagonism can address IBD by minimizing Th1, Th2, and Th17 responses, IFN production, and immune recruitment, consequently reducing inflammation and fibrosis. The three anti-TL1A drug development programs that target UC and CD display immense potential and it is highly likely that anti-TL1A therapeutics may soon emerge as strong alternatives to anti-TNF medications.

### CD40/CD40L

#### Signaling mechanism

The interaction between CD40 and its cognate ligand CD40L serves as a crucial costimulatory immune checkpoint. Membrane-bound CD40 is expressed in B cells constitutively. CD40 expression in myeloid APCs such as monocytes, macrophages, and DCs is induced by activating signals such as bacterial lipopolysaccharides (LPS), advanced glycation end-products (AGEs), and high mobility group protein 1 (HMGB1), and enhanced by cytokines such as interleukin-18 (IL-18), granulocyte/macrophage colony-stimulating factor (GM-CSF), interleukin-3 (IL-3), and interferon-γ (IFN-γ) (Takahashi et al., 2013; Tang et al., 2021). CD40L is expressed in activated CD4^+^ T cells but not in resting cells. Signals such as TCR engagement, co-stimulation, and interleukin-12 (IL-12) enhance CD40L expression in Th1, Th2, Th17, and T follicular helper (T_FH_) cells, as well as in CD8^+^ T cells and Tregs to a lesser extent (Tang et al., 2021; Tay et al., 2017). The ligation of CD40 with CD40L activates the non-canonical NF-κB, MAPK, and PI3K/AKT pathways by recruiting TRAF-1, -2, -3, -5 and -6, thereby initiating proinflammatory action through NF-κB and AP-1 transcription factors (Seibold and Ehrenschwender, 2015). CD40 signaling in B cells is implicated in driving proliferation, activation, Ab class switching, affinity maturation through somatic hypermutation, and differentiation into plasma and memory cells (Koike et al., 2019). CD40 activation in APCs such as DCs, monocytes, and macrophages is important for effective antigen presentation, inflammatory cytokine production, and T cell stimulation. Notably, CD40L–CD40 interactions between CD4^+^ T cells and DCs are imperative for DC licensing, which drives the priming and proliferation of CD8^+^ T cells for a robust T cell response in a CD40-dependent manner (Tay et al., 2017). Activated T cells also express CD40 with increased frequency and expression that correlates with the presence of autoimmune disease. CD40 signaling serves as a costimulatory cue for TCR-mediated CD4^+^ T cell activation and cytokine production (Munroe and Bishop, 2007) and mediates the generation of CD8^+^ T cell memory (Bourgeois et al., 2002).

#### Disease relevance

Polymorphisms in *CD40* are associated with psoriasis, UC, CD, RA, and SLE (Table 2), and CD40 is more abundant in UC colon and AD dermal samples compared to healthy controls (Dharmasiri et al., 2021). CD40/CD40L signaling is strongly implicated in SLE, RA, and IBD pathogenesis (Seibold and Ehrenschwender, 2015). In RA, both CD40L and CD40 are upregulated in diseased synovium from patients (Liu et al., 2001). Treatment of PBMC-derived macrophages from RA patients with anti-TNF agents resulted in lower CD40 expression (Degboé et al., 2019). Interestingly, synovial fibroblasts isolated from RA subjects display reduced TNF production when pretreated with anti-CD40 prior to coculture with synovial fluid mononuclear cells, suggesting a complementary relationship between TNF and CD40/CD40L signaling in inflammation and disease propagation (Liu et al., 2001). In murine models of CIA, CD40 antagonism attenuates inflammation, immune infiltration, cartilage, and bone erosion, and reduces serum Ab titers to collagen (Durie et al., 1993).

In SLE patients, serum levels of soluble CD40L are elevated, correlating strongly with disease activity (Goules et al., 2006). Membrane-bound CD40L is also more abundant in resting and activated T cells derived from SLE patients compared with healthy controls (Daoussis et al., 2007). Interestingly, B cells isolated from SLE patients spontaneously produce antibodies in a CD40L-dependent manner, suggesting a role for CD40L in autoimmunity (Grammer et al., 2003). In mouse models of chronic colitis, CD40L antagonism blocks IL-12 production, pathogenic Th1 response, and consequent inflammation, highlighting the promise of CD40/CD40L blockade in IBD (De Jong et al., 2000; Stuber et al., 1996).

#### Drug development

Elevated levels of circulating immune complexes composed of autoantibodies and self-antigens are observed in autoimmune conditions such as SLE. Platelets binding such immune complexes get activated, express CD40L, and induce differentiation, maturation, and type I IFN release in DCs, and consequently promote further autoantibody secretion by plasma cells (Duffau et al., 2010). While anti-CD40L showed promise in murine models of inflammatory conditions, notable past clinical trials involving anti-CD40L Ab therapies for autoimmune disorders have encountered thromboembolic complications stemming from platelet activation in response to the Fc domain on anti-CD40L drugs (Boumpas et al., 2003; Kawai et al., 2000; Langer et al., 2005). Mouse platelets lack FcγRIIa and are less susceptible to activation and aggregation by traditional anti-CD40L Ab treatment. Since autoimmune patients have elevated serum levels of soluble CD40L, treatment with Fc-domain intact anti-CD40L could form immune complexes consisting of soluble CD40L and anti-CD40L antibodies, which then activate platelets in an FcγRIIa (CD32)-dependent manner and trigger thromboembolic events (Langer et al., 2005; Robles-Carrillo et al., 2010). This discovery has prompted the development of safer anti-CD40/CD40L biologics with silenced Fc domains.

Given its immense potential, the inhibition of CD40/CD40L interaction is a focal point in multiple immunotherapies in development for conditions such as RA, SLE, and UC. Dapirolizumab pegol (BIIB133), a PEGylated F(ab’) fragment that targets CD40L, was assessed in a phase 2b study (NCT02804763) involving 182 SLE patients, where they received standard of care (SOC), along with either intravenous dapirolizumab administered every 4 wk at three doses (6, 24, and 45 mg/kg), or placebo. Dapirolizumab showed an acceptable safety profile and was well tolerated (Furie et al., 2021). While the trial failed to meet one of its primary objectives of establishing a relationship between drug dose and disease response, several disease activity scores and immune biomarkers showed improvements over placebo treatment at week 24 (Furie et al., 2021). During a post-treatment observational period, several disease activity scores remained unchanged. However, flares increased, and the levels of anti-dsDNA (double-stranded DNA), complement C3, and complement C4 returned to pathogenic levels seen at the start of the study, evidence of the inefficacy of SOC (Furie et al., 2021). These indications of a biological effect have spurred an ongoing phase 3 trial involving 321 patients (NCT04294667).

Frexalimab (SAR-441344) is a non-depleting anti-CD40L mAb with a modified Fc region that improves its safety by impairing binding to FcγRIIa and C1q, consequently attenuating platelet and complement activation, respectively. It is being investigated in a phase 2 trial recruiting 116 active SLE patients (NCT05039840). Iscalimab (CFZ533) is a human anti-CD40 mAb that is Fc-silent and therefore non-depleting. It is being investigated in a phase 2 study comprising 107 SLE patients (NCT03656562) and another recently completed phase 2 study involving 57 lupus nephritis (LN) patients whose results are yet to be posted (NCT03610516). It was previously determined to be safe in a phase 1 trial with 56 healthy volunteers and 20 RA patients with no thromboembolic events reported (Espié et al., 2020) (NCT02089087).

Dazodalibep (VIB4920/MEDI4920) is a non-antibody biologic formed as a fusion of human serum albumin with two fibronectin type III domains of tenascin-C (Tn3), a protein that can bind CD40L. It was evaluated in a phase 2 trial involving 78 adult participants with RA, with dosing on days 1, 15, 29, and 57 (NCT04163991). All four doses of dazodalibep elicited a significantly greater reduction in the disease activity relative to the placebo group (P < 0.05) at day 113, and the response was sustained until day 309 (Kivitz et al., 2023). AEs were comparable between the treatment and placebo groups, suggesting an acceptable tolerability profile. The absence of thromboembolic events in clinical trials is attributed to the lack of the Fc domain in this biologic (Kivitz et al., 2023). Additionally, an ongoing phase 2a study assesses the efficacy of dazodalibep combined with mycophenolate mofetil and prednisone in achieving a renal response in 74 participants with active LN (NCT05201469).

Abiprubart (KPL-404) is a CD40 antagonizing mAb that blocks CD40^–^CD40L interaction-mediated activation of B cells (Marken et al., 2021). Interim analysis of a phase 2 trial (NCT05198310) in 145 RA subjects with inadequate response or intolerance to at least one biologic disease-modifying antirheumatic drug (DMARD) or a Janus kinase inhibitor indicated that its primary endpoints were met (Kiniksa Pharmaceuticals, 2024). Ravagalimab (ABBV-323) is a CD40 antagonist mAb tested in a recently completed phase 2 trial in 42 UC patients (NCT03695185). The study was not placebo-controlled and only 18% of patients receiving the drug showed improvement in the Mayo endoscopic subscore at week 12.

Taken together, CD40/CD40L antagonism is intended to reduce type I IFN secretion, inhibit inflammatory cytokine production by myeloid cells, and mitigate autoantibody production by suppressing B cell activation and memory response, and may emerge as a therapeutic strategy for RA and SLE.

### OX40/OX40L

#### Signaling mechanism

OX40 (CD134, encoded by *TNFRSF4*) and its cognate ligand OX40L (CD252, encoded by *TNFSF4*) are well-studied costimulatory molecules facilitating T cell effector functions. OX40 is expressed on activated CD4^+^ and CD8^+^ T cells but not in naïve or resting memory T cells (Alves Costa Silva et al., 2020). OX40 expression in T cells is driven by TCR activation and is augmented by cytokines, including IL-1β, IL-2, and TNF, in addition to costimulatory interactions such as CD28 with B7 ligands and CD40L with CD40 (Webb et al., 2016). OX40/OX40L interactions are believed to occur in two stages and promote T cell effector functions (Fu et al., 2020). T cell costimulation through OX40 is initially mediated by OX40L expressed on APCs, including activated DCs and macrophages, in response to IFN-γ, prostaglandin E2, thymic stromal lymphopoietin (TSLP), and IL-18 exposure (Webb et al., 2016). Subsequent interactions between OX40^+^ T cells and OX40L expressed by activated B cells, NK cells, mast cells, and inflamed endothelial cells further promote T cell activation, expansion, and survival (Webb et al., 2016). Upon ligation by OX40L, the cytoplasmic tail of OX40 binds TRAF-2, -3, and -5 and causes activation of NF-κB and NFAT pathways and subsequent effector cytokine production (Alves Costa Silva et al., 2020). In human Tregs, OX40 signaling impairs suppressive functions (Jacquemin et al., 2018; Voo et al., 2013). Notably, in vivo experiments show that the activation of the OX40 pathway preferentially expands the antigen-specific T cell pool, and subsequently, these cells are resistant to immune suppression by Treg (Webb et al., 2016). In agreement, OX40 blockade using the antagonizing mAb amlitelimab significantly extended graft-versus-host disease (GvHD)-free survival and delayed clinical signs of acute GvHD in a nonhuman primate model by restraining CD4 T cell expansion and preserving Treg reconstitution following hematopoietic stem cell transplantation (Tkachev et al., 2017). Taken together, OX40 antagonism can be anti-inflammatory by inducing immune tolerance through Treg preservation and suppressing T cell memory differentiation and effector functions.

#### Disease relevance

GWAS studies have shown that *TNFSF4* is associated with AD, asthma, SLE, and RA (Table 2). Considering that IMIDs such as RA and UC are characterized by OX40/OX40L interactions occurring primarily between activated T cells and APCs within inflamed tissue (but not in peripheral blood cells), the antagonism of OX40 and OX40L represents a promising strategy for targeting areas of immune activity (Webb et al., 2016). Circulating Tregs from SLE patients exhibit elevated expression of OX40 and display impaired suppressive functions compared with healthy donors in an OX40L-dependent manner (Jacquemin et al., 2018). Further, Tregs colocalized with OX40L-expressing cells in active SLE skin lesions, implicating OX40 signaling for Treg suppression (Jacquemin et al., 2018). Similarly, there exist elevated soluble OX40L in psoriatic patient serum (Guo et al., 2019), increased number of OX40^+^ cells in psoriatic and AD lesions (Fujita et al., 2011; Guo et al., 2019), and strong correlations between AD disease activity scores and dermal OX40L abundance (Elsner et al., 2020).

While disruption of OX40/OX40L signaling holds promise for various IMIDs, substantial drug development efforts revolve around disorders such as AD and asthma that involve pathogenic type 2 immune responses (Nakahara et al., 2021). A positive feedback loop exists within the Th2 immune response and epidermal barrier dysfunction in AD. Th2-associated IL-4/IL-13 secretion decreases barrier proteins such as filaggrin and loricrin, leading to barrier disruption. Conversely, a compromised barrier promotes IL-25, IL-33, and TSLP secretions, which activate DCs and induce OX40L expression, consequently accelerating the Th2 response (Furue and Furue, 2021). In asthma, allergens induce epithelial cells and mast cells to secrete TSLP, triggering DC maturation and OX40L expression, consequently driving inflammation and Th2 response (Parnes et al., 2022). In murine models of skin and lung inflammation induced by intradermal and intranasal administration of TSLP and in an ovalbumin-induced allergic airway inflammation model, treatment with anti-OX40 Ab has consistently resulted in reduced inflammation (Seshasayee et al., 2007). Similarly, OX40 knockout mice display attenuated airway hyperreactivity, impaired Th2 response, 80–90% reduction in eosinophilia and mucus production, and lower goblet cell hyperplasia when primed with ovalbumin and challenged with intranasal administration of aerosolized antigen (Jember et al., 2001).

#### Drug development

Four recent drug development efforts are underway targeting OX40/OX40L in AD and asthma. Rocatinlimab (KHK4083/AMG451) is a fully human afucosylated anti-OX40 mAb that works by depleting OX40^+^ T cells. It is undergoing phase 3 trials in AD (NCT05398445 and NCT05651711 main studies), assessing monotherapy and combination therapy with topical corticosteroid and calcineurin inhibitors in adults and adolescents. The drug showed promising tolerance and efficacy data in a phase 2b study involving 274 patients with AD who displayed inadequate symptom relief with corticosteroids. Compared with the placebo at week 16 of the study that showed a −15% change in mean eczema area and severity index (EASI), a low dose treatment of 150 mg every 4 wk caused a −48.3% change, while a higher dose of 600 mg administered every 2 wk changed EASI by −57.4% (Guttman-Yassky et al., 2023). The study also reported sustained improvement in most patients after discontinuation of treatment. Notably, the drug showed good safety but not efficacy against psoriasis in a phase 1 study (Papp et al., 2017). These divergent results of rocatinlimab in AD and psoriasis may be explained by lower OX40L in psoriatic skin lesions compared with AD samples (Fujita et al., 2011).

Telazorlimab (ISB 830/GBR 830) is a humanized anti-OX40 mAb that underwent a phase 2b evaluation in 462 subjects with AD (NCT03568162). Subcutaneous administration of telazorlimab at 300 mg every 2 wk (following a loading dose of 600 mg) and at 600 mg every 2 wk (following a loading dose of 1,200 mg) resulted in significant improvement in EASI at 16 wk (−54.4% versus −34.2% placebo, P = 0.008 and −59.0% versus −41.8% placebo, P = 0.008, respectively) (Rewerska et al., 2023). Significant clinical improvement was maintained through 66 wk of follow-up, and the treatment was safe and well-tolerated (Rewerska et al., 2023).

Amlitelimab (KY1005/SAR445229) is a non-depleting human anti-OX40L mAb that blocks OX40L on APCs instead. In AD patients, it causes a decrease in the serum levels of IL-22 and IL-13, cytokines associated with Th22 and Th2 responses, respectively, in the underlying AD immunopathogenesis (Weidinger et al., 2022, 2023b). Interim results from an ongoing phase 2b trial with 390 AD patients (NCT05131477) showed that while all four dosage regimens of amlitelimab tested produced significant improvements in EASI from baseline to week 16 from placebo, the group that received 250 mg subcutaneously every 4 wk following a 500 mg loading dose showed the best improvement in EASI (61.5%) compared with placebo (29.4%, P < 0.0001) (Weidinger et al., 2023a). EASI-75 response achieved by week 24 of treatment was maintained in 69.2% and 58.5% of patients who continued treatment or withdrew from treatment for a further 28 wk, respectively (Sanofi, 2024). Recruitment for several phase 3 trials in AD patients are ongoing (NCT06130566, NCT06181435, NCT06241118, and NCT06224348). Additionally, a phase 2 dose-ranging study of amlitelimab is currently underway in asthma (NCT05421598).

IMG-007 is a humanized anti-OX40 IgG1 mAb featuring an engineered Fc region that extends its half-life and reduces Ab-dependent cellular cytotoxicity (ADCC) (Shen et al., 2023). Its safety and efficacy are being assessed in a phase 1b/2a study involving 55 patients with AD (NCT05984784).

In summary, OX40/OX40L blockade can regulate effector and memory T cell expansion and downregulate inflammation caused by Th1, Th2, Th17, and Th22 cells. It can also lower cytokine production and restore homeostasis between proinflammatory and anti-inflammatory T cells. Data from clinical studies strongly indicate OX40/OX40L inhibition as a potent and safe option for treating type 2 inflammatory diseases such as AD and asthma.

### BAFF/BAFF-R

#### Signaling mechanism

B cell activating factor (BAFF, encoded by *TNFSF13B*), alternatively B lymphocyte stimulator, is a costimulatory cytokine governing B cell survival, maturation, and IgG class switching and production (Batten et al., 2000; Castigli et al., 2005; Litinskiy et al., 2002). It binds to three receptors: BAFF receptor (BAFF-R, encoded by *TNFRSF13C*), transmembrane activator and CAML interactor (TACI), and B-cell maturation antigen (BCMA). BAFF is expressed by innate immune cells, such as monocytes, macrophages, neutrophils, and DCs when induced by TLR agonists and type I and II IFN (Manetta et al., 2014). BAFF is also produced by germinal center resident T_FH_ and follicular DCs, where both cell types play essential roles in stimulating B cell proliferation and differentiation (Zhang et al., 2005, 2015). BAFF-R, the dominant BAFF receptor in naïve B cells exiting the bone marrow, increases in abundance initially as B cells differentiate. BAFF-R expression subsequently decreases in B cells during differentiation to plasma cells, corresponding to the induction of TACI and BCMA expression (Zhang et al., 2005). The membrane-bound form of BAFF gets proteolytically cleaved by Furin, forming soluble active homotrimers that can activate BAFF-R, or the more potent but lowly abundant 60-mer complexes that are necessary for TACI activation (Bossen et al., 2008; Manetta et al., 2014). Ligation of BAFF-R by BAFF results in TRAF-2, -3, and -6 recruitment, and subsequent non-canonical NF-κB signaling (Hildebrand et al., 2010; Thompson et al., 2016). BAFF-R expression is primarily restricted to the B-cell lineage where its activation by BAFF ligation drives B cell maturation and proliferation.

#### Disease relevance

BAFF/BAFF-R signaling plays a key role in pathogenesis and disease progression of autoimmune diseases like RA and SLE. While RA patients exhibit elevated BAFF-R expression in synovial tissue (Woo et al., 2011), BAFF is upregulated in several IMIDs including, IBD (Zhang et al., 2016), RA (Ohata et al., 2005; Woo et al., 2011), and SLE (Stohl et al., 2003). The elevated levels of serum BAFF in SLE patients are associated with increased anti-dsDNA levels (Zhang et al., 2001) and SLE disease activity (Petri et al., 2008), and may be driven by *TNFSF13B* variants associated with SLE risks (Steri et al., 2017; Theodorou et al., 2018). Pathological inflammation in SLE is mediated by increased IFN-γ production by T cells, which induces BAFF production in monocytes/macrophages and the ensuing B cell activation and maturation through BAFF/BAFF-R signaling (Harigai et al., 2008). In RA patients, synovial expression of BAFF is elevated (Tan et al., 2003). Serum BAFF levels in RA patients positively correlate with bone destruction and other classic marks of RA, such as C-reactive protein (CRP), rheumatoid factor, disease activity score, swollen joint incidence, and X-ray scores (Zhang et al., 2021a). Therefore, antagonizing the BAFF/BAFF-R axis is an attractive therapeutic approach to autoimmune inflammatory diseases.

#### Drug development

Belimumab is a human IgG1 anti-BAFF mAb that, in 2011, became the first biologic drug approved by the United States Food and Drug Administration (FDA) for use in SLE patients with active, autoantibody-positive disease already on standard therapy (Dubey et al., 2011). It works by inhibiting the survival of B cells, including autoreactive B cells, and hampering their differentiation to plasma cells. In two phase 3 trials, concomitant SOC and Belimumab treatment met their primary efficacy endpoints of significantly higher SLE responder index (SRI) at week 52 (43.2% versus 33.5% in placebo, P = 0.017) (Furie et al., 2011; Navarra et al., 2011) (NCT00424476, NCT00410384). In these studies, belimumab administration reduced patient serum levels of IgG and autoantibodies, including anti-dsDNA, and normalized (increased) complement C3/C4 levels (Stohl et al., 2012). The drug also led to lower numbers of naïve, activated, and plasma B cells without affecting memory B cells or T cell populations (Stohl et al., 2012). In 2020, belimumab also became the first FDA-approved treatment for adult patients with active LN, following a pivotal phase 3 study (NCT01639339) that demonstrated its safety and efficacy. At week 104, patients receiving 10 mg/kg of belimumab intravenously in addition to the SOC achieved primary renal response (43% versus 32%, P = 0.03) and complete renal response (30% versus 20%, P = 0.02) at significantly higher rates than the placebo group (Furie et al., 2020). Interestingly, BAFF homeostasis disturbed by B cell depletion with rituximab (α-CD20) treatment results in the relapse of SLE with increased flares and anti-dsDNA in a manner that can be countered with combined belimumab treatment to prevent repopulation by autoreactive B cells (Atisha-Fregoso et al., 2021; Carter et al., 2013; Ehrenstein and Wing, 2016; Kraaij et al., 2021). These highlight the significance of disrupting BAFF signaling in lupus therapy. It is worth noting that belimumab failed to clearly demonstrate efficacy in a phase 2 evaluation involving 283 RA patients (NCT00071812). Clinical response was significantly higher with 1 mg/kg belimumab than placebo (34.7% versus 15.9%, P = 0.010), but not in patients administered 4 mg/kg (P = 0.068) or 10 mg/kg (P = 0.080) of the drug (Stohl et al., 2013).

Ianalumab (VAY736) is a BAFF-R antagonizing human IgG1 mAb that works by causing lysis of B cells through ADCC and the blockade of BAFF-R (Bowman et al., 2022). In a phase 2 study (NCT03656562) involving 67 SLE patients who received 300 mg subcutaneous ianalumab every 4 wk for 28 wk, interim results show that the drug was well tolerated, and the study met its primary endpoint on clinical response. Ianalumab treatment lowered flares and autoantibody titers, depleted CD19^+^ B cells and normalized C3/C4 levels (Cuker et al., 2023; Dörner et al., 2024) (Shen, N., A. Gordienko, J.C. Hernández, N. Agmon-Levin, P. Narongroeknawin, K. Romanowska-Prochnicka, H. Ciferska, M. Kodera, J. Wei, and P. Leszczynski. 2023. *Arthritis Rheumatol.* Abstr. 2487). Two phase 3 studies in SLE patients (NCT05639114, NCT05624749) and one phase 3 study in LN subjects (NCT05126277) have been initiated.

Rozibafusp alfa (AMG570) is a bispecific Ab that works by concurrently antagonizing BAFF to reduce circulating naïve B cells and ICOSL to reduce APC (DC and B cell)-mediated T cell activation and proliferation (Lei et al., 2020). A phase 2b study in 244 participants with active SLE assessing the efficacy of three different doses of rozibafusp alfa at week 52 has been completed and results are awaited (NCT04058028).

Telitacicept (RC18) is an Fc fusion of the extracellular domain of TACI capable of neutralizing two ligands of the TACI receptor, BAFF, and a proliferation-inducing ligand (APRIL). In a phase 3 trial in 335 SLE patients (NCT04082416), weekly administration of 160 mg of telitacicept plus SOC for 52 wk achieved a significantly higher SRI-4 response rate (82.6%, P < 0.001) than the placebo plus SOC treatment group (38.1%) (Wang et al., 2023; Wu et al., 2022). The drug had a safety profile similar to that of the placebo group and evoked a sustained increase in C3/C4 levels and a decrease in IgM, IgG, IgA, and CD19^+^ B cells (Wang et al., 2023). It received conditional marketing approval for treating active, autoantibody-positive, adult SLE patients in 2021 in China, and a fast-track designation from the FDA in 2020 for SLE treatment (Fan et al., 2022). A recent global follow-up phase 3 study of telitacicept efficacy in SLE was initiated (NCT05306574). In addition, telitacicept is being evaluated in a phase 3 trial in 480 participants with RA who exhibit inadequate response to methotrexate therapy (NCT03016013). A molecule that also neutralizes BAFF and APRIL, povetacicept (ALPN-303), is a Fc fusion with TACI that is engineered for enhanced affinity to its targets. It entered a phase 1b/2a study in 56 patients with autoimmune kidney diseases including LN (NCT05732402) and is destined for a phase 2 SLE study (Alpine Immune Sciences, 2024; Evans et al., 2023).

Together, BAFF/BAFF-R antagonism moderates plasma B cells, lowers autoreactive antibodies and immune complexes in circulation, and effectively combats systemic inflammation and tissue damage in SLE (including LN). Despite the promise of BAFF/BAFF-R inhibition in other rheumatic diseases including RA, clear benefits have not yet been established.

## Opportunities and challenges in TNF/TNFR drug therapy for inflammatory diseases

In this section, we highlight the various limitations of current approved medications and the opportunity for novel therapeutics including small molecule inhibitors and combination treatments. We also summarize promising drug development activities that may result in a wider array of IMID medications.

### Limitations of the approved anti-TNF biologics necessitate drug development

The TNF antagonist biologics that are currently approved for use in various IMIDs improve clinical outcomes and quality of life for patients. Despite this, these drugs suffer from high primary non-response rates (PNR). In CD patients, the initial induction with anti-TNF treatment results in a PNR ranging from 20% to 40% during trials, a further loss of response (LOR) in 23–46% of patients over the 12 mo post-anti-TNF initiation, and treatment discontinuation in 7–25% of patients (Ben-Horin et al., 2014). Factors that affect response to TNF antagonism in IBD include duration of disease, small bowel involvement, CRP levels, smoking status, and incidence of mutation in apoptosis genes such as *FASLG* and *CASP9* (Siegel and Melmed, 2009). In the various clinical trials involving RA patients, anti-TNF biologics administered along with methotrexate have achieved ACR50 response (50% improvement in a standard set of measures of disease) in only 18–48% of participants by 6 mo (Johnson et al., 2019). The variability in the effectiveness of biologics in RA treatment can be attributed to the differences in the baseline characteristics of patients and their prior drug experience. For instance, in the various clinical trials of RA patients with anti-TNF agents, ACR20 response rates are generally higher in methotrexate-naïve subjects than in methotrexate-experienced or TNF inhibitor-experienced subjects (Smolen and Aletaha, 2015). LOR to anti-TNF therapy is usually addressed by dose escalation, anti-TNF drug substitution, or switch to a different class of drug. Other drawbacks of anti-TNF biologics include increased risk for the development of opportunistic infections (for instance, latent tuberculosis reactivation), immunogenicity that may result in anti-drug antibody (ADA) formation and progressive efficacy loss, induction of autoantibodies (including antinuclear antibodies and anti-dsDNA) that may trigger autoimmune conditions in rare cases, and potentially higher risk for specific malignancies (such as lymphomas) (Atzeni et al., 2013; Kalliolias and Ivashkiv, 2016). Overall, these highlight the limitations of the current anti-TNF therapeutics and the need for new and better medications.

Biologics possess high affinity and specificity toward their targets, but their molecular size limits oral bioavailability. Oral biologics represent an attractive prospect that allows for non-invasive delivery, non-immunogenicity, and lower production costs, but efforts thus far have been unsuccessful. One recent example is V-565, a protease-resistant biologic based on an anti-TNF immunoglobulin chain variable domain that was in development for CD treatment. Preclinical trials demonstrated its survival in the intestinal tract of cynomolgus monkeys, and its TNF-neutralizing potency and cytokine inhibitory capabilities match that of adalimumab and infliximab, respectively (Crowe et al., 2018, 2019). In a phase 2 study involving 125 participants with active CD, V-565 administered thrice daily for 6 wk was found to be safe and well-tolerated (NCT02976129). However, it did not cause clinical remission at a greater rate than placebo.

### Small-molecule inhibitors of TNF-TNFR interactions hold promise as novel therapeutics

Small molecules are simpler to manufacture and carry lower risks from immunogenicity compared to biologics. Their oral bioavailability also makes them an attractive therapeutic modality, especially for chronic conditions. One of the first described small molecule TNF inhibitors, SPD-304, destabilizes the TNF trimer by displacing one of the subunits, causing its dissociation into TNF dimers, and thereby restricting TNF/TNFR1 binding and signaling (He et al., 2005). Despite SPD-304 suffering from metabolic instability, toxicity, non-selective binding to TNF, and poor solubility, hampering further development (Mascret et al., 2021), there have been several efforts to identify more molecules that work through the same modality (Sun et al., 2020; Wang et al., 2022). JNJ525 is one such molecule that aggregates into assemblies, competes for, and displaces a subunit of the TNF trimer, causing a change in its quaternary structure, consequently forming dimers of TNF dimers, and hindering receptor engagement (Blevitt et al., 2017). TIM1 is another lead compound discovered by applying in silico screening, based on the crystal structure of SPD304-bound TNF (Javaid et al., 2022). TIM1 and its analog TIM1c disrupt TNF homotrimerization by binding to the central hydrophobic cavity of the monomeric form, enabling them to disrupt TNF signaling, and attenuate inflammation and apoptosis. In a murine CIA model, TIM1c reduced arthritis index, paw swelling, histological indicators of pathology, and immune infiltration, exhibiting efficacy comparable to etanercept administration (Javaid et al., 2022).

UCB-9260 is a novel small molecule discovered through fragment-based screening and crystallography-guided optimization that compromises TNF/TNFR1 interactions and signaling (O’Connell et al., 2019). Allosteric binding by this small molecule does not cause conformational changes. Instead, it binds entirely within the core of the TNF trimer and stabilizes it in an asymmetric naturally occurring open conformation through conformational selection, resulting in distortion at one of the TNFR1 binding sites (McMillan et al., 2021). Interestingly, it is believed that this asymmetric trimer conformation would hold stable without a bound inhibitor, owing to a significant free-energy barrier to return to the more stable symmetric closed conformation (O’Connell et al., 2019). This novel inhibitory mechanism has spurred efforts to discover more small molecules that stabilize the asymmetric conformation of the TNF trimer through allostery. Applying scaffold hopping and structure-based drug design principles to the benzimidazole-based scaffold of the UCB-9260 molecule led to the development of compound 42 (Xiao et al., 2020). Efficacy testing of compound 42 in a murine collagen antibody-induced arthritis model revealed that it achieves a dose-dependent reduction in clinical scores, inflammatory cytokine levels, and leukocyte cell surface receptor expression that was comparable to treatment with etanercept (Xiao et al., 2020). Compound 12 is another small molecule that causes the allosteric desymmetrization of the TNF trimer that was identified by fragment-based drug discovery (Dietrich et al., 2021). In a murine glucose-6-phosphate isomerase-induced arthritis model, compound 12 administration led to significantly lower paw swelling, and the efficacy was comparable to anti-TNF treatment (Dietrich et al., 2021). SAR441566 is the most promising small molecule inhibitor of TNF and a derivative of UCB-9260. As described in the previous section, its anti-inflammatory properties stem from its ability to stabilize an asymmetric conformation of the TNF trimer, and it is under phase 2 investigations for the treatment of RA and plaque psoriasis (McMillan et al., 2021; Vugler et al., 2022).

Small molecules inhibiting other TNF/TNFR targets are also of potential interest. BIO8898 is a synthetic inhibitor of CD40L that intercalates between two subunits of the homotrimer form of CD40L, breaking its symmetry and causing allosteric disruption at two out of three of its receptor binding sites (Silvian et al., 2011). BIO8898 limited CD40L/CD40 signaling and apoptosis in preclinical studies (Silvian et al., 2011). A few other studies have also reported small molecules capable of disrupting CD40/CD40L interactions (Bojadzic et al., 2018; Chen et al., 2017), indicative of the abundant interest in developing an optimized CD40/CD40L inhibitor. While TNF/TNFR inhibitors that disrupt protein–protein interactions are challenging to design, such molecules represent excellent promise and opportunity for IMID therapy. It is to be noted that small molecule inhibitors of TNF/TNFR proteins may still suffer from some of the same limitations plaguing biologic therapeutics, i.e., low responder rates, and opportunistic infection and malignancy risks.

### TNF/TNFR superfamily members show promise as combinatorial targets

To overcome the limitations of current IMID therapies, combinations are commonly employed in the clinic, particularly to treat refractory patients. Generally, they take the form of anti-TNF biologics paired with other traditional systemic drugs to expedite response (e.g., with glucocorticoids) or to improve efficacy (e.g., immunogenicity deterrence with immunosuppressants). Drug development efforts are ongoing to test if combinations of two biologics with distinctive mechanisms of action can act with added efficacy and robustness. A recent phase 2a trial evaluated the combined efficacy of two approved biologics (JNJ-78934804): the anti-TNF drug golimumab and the anti-IL-23 mAb guselkumab in 214 patients with UC (NCT03662542). While guselkumab is indicated for psoriasis and psoriatic arthritis, it has demonstrated efficacy in phase 2 studies for UC (Dignass et al., 2022) (NCT04033445) and CD (Sandborn et al., 2022) (NCT03466411) treatment. At week 12, a greater proportion of patients receiving the combination agents (83%) achieved clinical response compared with golimumab (61%, P = 0.0032) and guselkumab (75%, P = 0.2155) monotherapy, necessitating validation through larger trials (Feagan et al., 2023b). The combination of these biologics (JNJ-78934804) is also being evaluated in more extensive phase 2b trials for the treatment of CD (NCT05242471) and UC (NCT05242484). The combination of nipocalimab and certolizumab is another instance of two biologics being tested in combination. Nipocalimab is a neonatal Fc receptor (FcRn) antagonist that can lower circulating IgG levels, including that of pathogenic autoantibodies responsible for autoimmune disease. It is being tested as a monotherapy in phase 2 clinical trials for RA (NCT04991753) and SLE (NCT04882878), and in combination with the anti-TNF drug certolizumab for RA (NCT06028438). α-TNF treatment has also been tested in combination with IL-17 neutralization with mixed results. Bimekizumab, a dual inhibitor of IL-17A/IL-17F approved for psoriasis, was efficacious when combined with certolizumab in RA patients who initially (8 wk) displayed inadequate response to certolizumab alone in a phase 2a trial (NCT02430909) and reduced disease activity levels on-par with responders on certolizumab alone by week 44 (Glatt et al., 2019). In contrast, ABT-122, a TNF/IL-17 bispecific neutralizer, failed to show superiority to adalimumab treatment in α-TNF-naïve RA patients (Genovese et al., 2018).

Combination therapies may also drill down on two targets that share pathways to perturb signaling crosstalk and restrain pathological axes spanning multiple cell types. Additionally, the modulation of two molecules belonging to the same signaling cascade, where one is downstream of the other, bestows benefits from additive efficacy and reduced failure rates. An example is tibulizumab (ZB-106/LY-3090106), an anti-BAFF and anti-IL-17A bispecific antibody (Benschop et al., 2019). In addition to activating B cells, BAFF can also promote the expansion of Th17 cells which are a major source of the pro-inflammatory cytokine IL-17. Tibulizumab showed promise in preclinical studies by demonstrating potent neutralization of BAFF and IL-17 (Benschop et al., 2019) and was previously considered for the treatment of RA (NCT01925157). Phase 2 studies are being planned for other inflammatory diseases including hidradenitis suppurativa (Zura Bio Ltd., 2023). SAR-443726 is a novel anti-OX40 and anti-IL-13 bispecific nanobody that was under development to mitigate type 2 immunity underlying conditions such as AD. Here, the rationale is to curb signaling through OX40 that promotes the generation and maintenance of Th2 cells and by IL-13 secreted by Th2 cells that can trigger activation of ILC2 cells. PF-07261271 is an α-TL1A and α-IL-12p40 bispecific antibody that is under development for IBD treatment (NCT05536440). A combined blockade of TL1A and IL-12/IL-23 signaling is hypothesized to limit their synergy, consequently downregulating IFN-γ and IL-17 production by CD4^+^ T cells and attenuating Th1/Th17 activation that underlies IBD (Takedatsu et al., 2008). Clinical development is also underway to capitalize on the synergy that exists between two significant disease-driving cytokines, TNF and IL-6. It is hypothesized that combinatorial blocking of these cytokines may have anti-inflammatory effects on two of the effector cell types in RA, fibroblasts, and CD4^+^ T cells (Biesemann et al., 2023). In agreement, an anti-TNF and anti-IL-6 bispecific nanobody demonstrated superior anti-inflammatory effects versus monospecific comparators in fibroblast-like synovial cell/T cell cocultures (Biesemann et al., 2023).

Preclinical studies have demonstrated proinflammatory synergies between TNF/TNFR signaling cascades that may be targeted to curb disease progression. In a murine asthma model, it has been shown that combinatorial antagonism of OX40L and CD30L inhibits the proliferation of effector memory T cells and protects test animals from allergic airway inflammation (Gracias et al., 2021). Monotherapy with the individual blocking Ab had a negligible effect on inflammation, suggesting synergy between the two co-stimulatory molecules, and the necessity for dual blockade therapy. Several studies prior have also revealed the synergetic roles of OX40 and CD30 signaling in T cell memory related to infections and autoimmune conditions (Gaspal et al., 2008; Gaspal et al., 2005; Gaspal et al., 2011; Withers et al., 2009). Synergy has also been reported between RANKL and TNF in osteoclast activation, and the dual blockade of these signaling molecules has been shown to reduce bone erosion and cartilage damage in a murine transgenic human TNF model (Fuller et al., 2002; Zwerina et al., 2004). TNF-like weak inducer of apoptosis (encoded by *TNFSF12*) has also been reported to synergize with TNF to upregulate signature genes in psoriasis, where dual antagonism of these two cytokines results in a lower number of proliferating keratinocytes (Gupta et al., 2021).

A rational search for combinatorial targets within the TNF/TNFR superfamilies may be justified for two more reasons. First, there is substantial cross-reactivity between the ligands and receptors of these superfamilies, that may drive synergy. Second, as both a consequence and mediator of inflammation, TNF may be responsible for inducing the expression of several of the TNF/TNFR co-stimulatory molecules. Concomitant aberrant signaling from several TNF/TNFR proteins may amplify inflammation and drive IMIDs, indicating opportunity from combination targets within these superfamilies. An example of clinical trials involving combination biologics is the phase 2 evaluation of dazodalibep, a CD40L inhibitor, in combination with anti-TNF medications (etanercept or adalimumab) is underway in RA patients with inadequate response to anti-TNF treatment (NCT05306353). Several bispecific Abs that target two pathogenic TNF/TNFR proteins are also under consideration. For instance, AMG-966 is a human aglycosylated bispecific Ab against TNF and TL1A that held promise for treatment of IBD. While preclinical assessments indicated minimal risk of immunogenicity, phase 1 trials showed that 98% of participants developed neutralizing ADA owing to the formation of large immunocomplexes of the drug with TNF and TL1A, and the consequent loss of exposure (Kroenke et al., 2021). Another noteworthy bispecific is SAR-442970, an anti-TNF and anti-OX40L nanobody whose immunomodulation efficacy is being assessed in a phase 2 trial involving patients with hidradenitis suppurativa (NCT05849922).

While combination anti-TNF/TNFR therapeutics have the potential to improve clinical response rates over traditional therapeutics, such combinations should be approached with caution due to the potential for additive immunosuppression that affects both innate and adaptive immunity and increased vulnerability to opportunistic infections. It is crucial to carefully monitor patients under such treatments to manage and mitigate these potentially heightened infection risks effectively.

### Other promising TNF/TNFR targets against the focus inflammatory diseases

Given the immense importance of the TNF/TNFR superfamilies as pro-inflammatory and co-stimulatory immune checkpoint molecules, TNF/TNFR proteins other than the ones reviewed in Promising TNF/TNFR targets in inflammatory diseases section have also garnered interest as targets for antagonism in focus IMIDs. In the final subsection, we highlight promising early drug development focusing on such novel TNF/TNFR signaling axes.

#### CD30/CD30L

Signaling via CD30 (encoded by *TNFRSF8*) and reverse signaling through CD30L (encoded by *TNFSF8*) are major prosurvival and proinflammatory signals in activated T cells and professional APCs, respectively. CD30 expression is upregulated in several IMIDs, including RA, SLE, AD, and asthma (Oflazoglu et al., 2009), and CD30 polymorphisms are associated with eczema (Table 2), suggesting that CD30/CD30L signaling could be an important determinant of inflammatory processes in these diseases. MK-8690 (PRA-052) is a human anti-CD30L mAb that is under development for IBD treatment. A phase 1 trial is ongoing to test its safety and pharmacological attributes (NCT05603182).

#### 4-1BB

Cosignaling via 4-1BB (encoded by *TNFRSF9*) expressed on activated T cells promotes their survival, proliferation, and cytokine production. Whereas 4-1BB agonism is a therapeutic direction that is currently pursued for cancer treatment, a recently described chimeric antigen receptor (CAR) T and NK cell therapy uses an “alloimmune defense receptor” (ADR) to protect allogenic CAR cells from host rejection and immune-mediated elimination (Mo et al., 2021). FT522 is an off-the-shelf, induced pluripotent stem cell-derived CAR-NK cell immunotherapy that capitalizes on the transient 4-1BB upregulation in activated lymphocytes and uses its 4-1BB–selective ADR to target alloreactive T and NK cells and maintain functional persistence. The CAR-NK cells eliminate CD19 and CD20 expressing B cells, and this cell therapy is being pursued for the treatment of B cell lymphoma in a phase 1 safety and tolerability assessment (NCT05950334). Importantly, preclinical retooling of 4-1BB–specific ADR-expressing cell therapies to orchestrate the selective elimination of pathogenic lymphocytes and induce immunotolerance in autoimmune conditions and GvHD has been reported (Fate Therapeutics Inc., 2023).

#### Receptor activator of nuclear factor κ-Β (RANK)/RANK ligand (RANKL)

Signaling originating from membrane-bound RANKL (encoded by *TNFSF11*) expressed by osteoblasts is responsible for myeloid cell differentiation and maturation into osteoclasts via interactions with RANK (encoded by *TNFRSF11A*). RANK/RANKL signaling is a major driver of bone resorption and homeostasis, making it an ideal target for antagonism to treat osteoporosis. Denosumab (AMG 162), a human anti-RANKL mAb that is FDA-approved for the treatment of osteoporosis and certain bone metastases, is additionally approved for use in Japan to suppress bone erosion associated with RA. In a phase 3 study involving RA patients receiving conventional synthetic DMARDs, 654 patients received 60 mg denosumab subcutaneously every 3 or 6 mo, or placebo (NCT01973569). There was significantly lower progression of joint destruction with denosumab treatment, and the mean change in total sharp score at 12 mo from baseline was 0.72 (P = 0.0055) and 0.99 (P = 0.0235) in the patients receiving denosumab every 3 and 6 mo, respectively, compared with 1.49 in the placebo group (Takeuchi et al., 2019). However, no significant differences in the joint space narrowing score were achieved with denosumab treatment.

#### BCMA

BAFF and APRIL (encoded by *TNFSF13*) bind to BCMA (encoded by *TNFRSF17*), a paralog of BAFF-R, to drive plasma cell survival. Their preferential expression on mature B cells, specifically, long-lived bone marrow plasma cells and plasmablasts is being capitalized in several plasma cell–depleting CAR-T therapies that are under development for SLE treatment. Descartes-08 is a phase 2 evaluation in 30 SLE patients to study RNA-based autologous BCMA-specific CAR-T therapy for drug safety and manufacturing feasibility (NCT06038474). Another example is CD19-BCMA compound CAR-T therapy that eliminates autoantibodies by dual targeting of CD19 on B cells (aimed at preventing the accumulation of autoreactive plasma cells) and BCMA on long-lived plasma cells (Zhang et al., 2021b). In phase 1 proof-of-concept evaluations involving 13 LN patients, the treatment was well tolerated, and all patients achieved significant symptom and medication-free remission, tested negative for autoantibodies, and displayed normal complement levels at 3 mo (Wang et al., 2024; Yuan et al., 2024) (NCT04162353 and NCT05474885). GC-012F is another autologous CAR-T cell therapy directed towards BCMA and CD19. Recruitment is ongoing for two phase I studies in refractory SLE patients to evaluate the safety and determine the dose for phase 2 trials (NCT05858684 and NCT05846347).

## Concluding remarks

Dysregulated TNF/TNFR signaling is a characteristic feature of several IMIDs, and their antagonism represents a very promising avenue for disease remission. Despite their limitations, current anti-TNF and anti-BAFF biologics have been clinical successes for IMID management since their first approvals over a decade ago. Nonetheless, significant interest lies in improving the efficacy and response rates of current TNF antagonists. In addition, genetic, transcriptomic, and clinical evidence implicate further TNF/TNFR family members in IMIDs, driving drug development efforts that seek to remedy SLE/RA, AD, and IBD by blocking aberrant signaling involving CD40/CD40L, OX40/OX40L, and TL1A, respectively. While mAbs and soluble fragments of transmembrane receptors are classic approaches for target antagonism, several innovative approaches are being developed that target the TNF/TNFR proteins. For instance, ADCs improve selectivity of drug action, and small molecules allow for non-invasive administration and lower immunogenicity. Evidence of synergy involving TNF/TNFR proteins suggests that combination therapies that curb two concomitant inflammatory signaling molecules may also be beneficial in IMID management. Indeed, several drug development programs addressing IMIDs involve bispecific antibodies that simultaneously block two proteins that contribute to pathogenic signaling. In addition, the electrical stimulation of the vagus nerve shows promise in clinical trials as a non-drug anti-inflammatory treatment that can reduce systemic TNF levels and complement traditional IBD and RA therapeutics (D’Haens et al., 2023; Jin et al., 2024; Koopman et al., 2016; Sinniger et al., 2020) (NCT01569503 and NCT02311660).

Advances in biomarker and genetics research can potentially accelerate drug discovery and enhance patient response to TNF/TNFR blockade in IMIDs. Currently, clinical management of IMIDs involves cycling through different anti-TNF agents to address inadequate primary response and LOR. However, personalized medical advice could become feasible in the future through models based on patient genetics and disease characteristics predicting clinical response to various treatment options. For instance, biomarkers identified in subpopulations of IBD patients who were more responsive to anti-TL1A treatments are envisioned to drive precision medicine and guide physicians toward patient-specific treatment strategies with higher success rates (Hassan-Zahraee et al., 2022; Merck & Co. Inc., 2023; Roivant Sciences Ltd., 2023a). Since several IMIDs share immunological features and mechanisms, biomarkers associated with these diseases can also be used to better stratify patients and optimize clinical trials. Basket trials, involving patients with different IMIDs but common underlying mechanisms and molecular characteristics, may expedite drug evaluation for multiple conditions. Umbrella trials, on the other hand, stratify patients based on biomarkers within the same disease to test various therapeutics and identify optimal treatment approaches. While such master protocols are more prevalent in oncology trials, they can also be adopted to test more hypotheses and increase trial efficiency in immunology research (Grayling et al., 2021; Peeva et al., 2024), especially when pursuing combination targets within the TNF/TNFR superfamilies. Going forward, leveraging innovative solutions from high-dimensional biomarker data has the potential to enhance trial efficiency, improve patient outcomes, and inform disease management choices.

## Supplementary Material

Table S1 shows TNF/TNFR members and their attributes.

Table S2 shows select phase 3 clinical trials of FDA approved TNF-α antagonists and their results.
