# Supplementary material for: Targeting TNF/TNFR superfamilies in immune-mediated inflammatory diseases
Source: J Exp Med. 2024 Sep 19;221(11):e20240806. doi: 10.1084/jem.20240806 (PMC11413425; doi:10.1084/jem.20240806)
Supplement: Table S1 — shows TNF/TNFR members and their attributes. [file JEM_20240806_TableS1.docx]

**Table S1.** **TNF/TNFR members and their attributes.**

| Gene  (Product) | Synonyms | Description | Noteworthy canonical interactor(s) |
| --- | --- | --- | --- |
| Ligands  *LTA* (LT-α) | LT, TNFB, TNFSF1 | Lymphotoxin alpha | Homotrimer: TNFR1, TNFR2, HVEM. Heterotrimer: LTβR |
| *TNF* (TNF) | DIF, TNF-alpha, TNFA, TNFSF2 | Tumor necrosis factor | TNFR1, TNFR2 |
| *LTB* (LT-β) | p33, TNFC, TNFSF3 | Lymphotoxin beta | LTβR |
| *TNFSF4* (OX40L) | CD252, gp34, OX-40L, TXGP1 | TNF superfamily member 4 | OX40 |
| *CD40LG*  (CD40L) | CD154, CD40L, gp39, hCD40L, HIGM1, IMD3, TNFSF5, TRAP | CD40 ligand | CD40 |
| *FASLG* (FasL) | APT1LG1, CD178, FasL, TNFSF6 | Fas ligand | FAS, DcR3 |
| *CD70* (CD70) | CD27L, CD27LG, TNFSF7 | CD70 molecule | CD27 |
| *TNFSF8* (CD30L) | CD153, CD30LG | TNF superfamily member 8 | CD30 |
| *TNFSF9* (4-1BBL) | 4-1BB-L, 4-1BBL | TNF superfamily member 9 | 4-1BB |
| *TNFSF10* (TRAIL) | Apo-2L, CD253, TL2, TRAIL | TNF superfamily member 10 | TRAILR1, TRAILR2, TRAILR3, TRAILR4, OPG |
| *TNFSF11* (RANKL) | CD254, ODF, OPGL, RANKL, TRANCE | TNF superfamily member 11 | RANK, OPG |
| *TNFSF12* (TWEAK) | APO3L, DR3LG, TWEAK | TNF superfamily member 12 | TWEAKR |
| *TNFSF13* (APRIL) | APRIL, CD256 | TNF superfamily member 13 | TACI, BCMA |
| *TNFSF13B* (BAFF) | BAFF, BLYS, CD257, TALL-1, TALL1, THANK, TNFSF20 | TNF superfamily member 13b | TACI, BCMA, BAFFR |
| *TNFSF14* (LIGHT) | CD258, HVEM-L, LIGHT, LTg | TNF superfamily member 14 | HVEM, LTβR, DcR3 |
| *TNFSF15* (TL1A) | MGC129934, MGC129935, TL1, TL1A, VEGI, VEGI192A | TNF superfamily member 15 | DR3, DcR3 |
| *TNFSF18* (GITRL) | AITRL, GITRL, TL6 | TNF superfamily member 18 | GITR |
| *EDA* (EDA) | ED1, ED1-A1, ED1-A2, EDA-A1, EDA-A2, EDA1, EDA2, HED, ODT1, XHED, XLHED | Ectodysplasin A | EDAR, XEDAR |
| Receptors  *TNFRSF1A* (TNFR1) | CD120a, TNF-R, TNF-R-I, TNF-R55, TNFAR, TNFR1, TNFR60 | TNF receptor superfamily member 1A | TNF, LT-α. Also associates with TRAF2, and TRADD. |
| *TNFRSF1B* (TNFR2) | CD120b, p75, TNF-R-II, TNF-R75, TNFBR, TNFBR, TNFR80 | TNF receptor superfamily member 1B | TNF, LT-α. Also associates with TRAF1, and TRAF2. |
| *LTBR* (LTβR) | D12S370, TNF-R-III, TNFCR, TNFR-RP, TNFR2-RP, TNFRSF3 | Lymphotoxin beta receptor | LIGHT and the heterodimer of LT-α and LT-β. Also associates with TRAF3, TRAF4 and TRAF5. |
| *TNFRSF4* (OX40) | ACT35, CD134, OX40, TXGP1L | TNF receptor superfamily member 4 | OX40L. Also associates with TRAF2, TRAF3 and TRAF5. |
| *CD40* (CD40) | Bp50, p50, TNFRSF5 | CD40 molecule | CD40L. Also associates with TRAF1, TRAF2, TRAF3, TRAF5 and TRAF6. |
| *FAS* (Fas) | APO-1, APT1, CD95, FAS1, TNFRSF6 | Fas cell surface death receptor | FasL |
| *TNFRSF6B* (DcR3) | DcR3, M68, TR6 | TNF receptor superfamily member 6b | Neutralizes FasL, LIGHT, and TL1A. |
| *CD27* (CD27) | S152, TNFRSF7, Tp55 | CD27 molecule | CD70. Also associates with TRAF2. |
| *TNFRSF8* (CD30) | CD30, D1S166E, KI-1 | TNF receptor superfamily member 8 | CD30L. Also associates with TRAF1, TRAF2, TRAF3, and TRAF5. |
| *TNFRSF9* (4-1BB) | 4-1BB, CD137, ILA | TNF receptor superfamily member 9 | 4-1BBL. Also associates with TRAF1, TRAF2, and TRAF3. |
| *TNFRSF10A* (TRAILR1) | Apo2, CD261, DR4, TRAILR-1, TRAILR1 | TNF receptor superfamily member 10a | TRAIL. Also associates with FADD and TRADD. |
| *TNFRSF10B* (TRAILR2) | CD262, DR5, KILLER, TRAIL-R2, TRAILR2, TRICK2A, TRICKB | TNF receptor superfamily member 10b | TRAIL. Also associates with FADD and TRADD. |
| *TNFRSF10C* (TRAILR3) | CD263, DcR1, LIT, TRAILR3, TRID | TNF receptor superfamily member 10c | Neutralizes TRAIL |
| *TNFRSF10D* (TRAILR4) | CD264, DcR2, TRAILR4, TRUNDD | TNF receptor superfamily member 10d | Neutralizes TRAIL |
| *TNFRSF11A* (RANK) | CD265, FEO, LOH18CR1, PDB2, RANK | TNF receptor superfamily member 11a | RANKL. Also associates with TRAF1, TRAF2, TRAF3, TRAF5, and TRAF6. |
| *TNFRSF11B* (OPG) | OCIF, OPG, TR1 | TNF receptor superfamily member 11b | Neutralizes RANKL, and TRAIL |
| *TNFRSF12A* (TWEAKR) | CD266, FN14, TweakR | TNF receptor superfamily member 12A | TWEAK. Also associates with TRAF1, TRAF2, and TRAF3. |
| *TNFRSF13B* (TACI) | CD267, IGAD2, TACI | TNF receptor superfamily member 13B | APRIL and BAFF. Also associates with TRAF2, TRAF5, and TRAF6. |
| *TNFRSF13C* (BAFF-R) | BAFFR, CD268 | TNF receptor superfamily member 13C | BAFF. Also associates with TRAF2, TRAF3, and TRAF6. |
| *TNFRSF14* (HVEM) | ATAR, CD270, HVEA, HVEM, LIGHTR, TR2 | TNF receptor superfamily member 14 | LIGHT, LT-α. Also associates with TRAF2, TRAF3, and TRAF5. |
| *NGFR* (NGFR) | CD271, p75NTR, TNFRSF16 | Nerve growth factor receptor | No TNF ligand. Associates with TRAF2, TRAF4, and TRAF6. |
| *TNFRSF17* (BCMA) | BCM, BCMA, CD269, TNFRSF13A | TNF receptor superfamily member 17 | BAFF and APRIL. Also associates with TRAF1, TRAF2, TRAF3, TRAF5, and TRAF6. |
| *TNFRSF18* (GITR) | AITR, CD357, GITR | TNF receptor superfamily member 18 | GITRL. Also associates with TRAF1, TRAF2, and TRAF3, but not TRAF5 and TRAF6. |
| *TNFRSF19* (TROY) | TAJ, TAJ-alpha, TRADE, TROY | TNF receptor superfamily member 19 | No TNF ligand. Associates with TRAF1, TRAF2, TRAF3 and TRAF5. |
| *TNFRSF21* (DR6) | CD358, DR6 | TNF receptor superfamily member 21 | No TNF ligand. Associates with TRADD. |
| *TNFRSF25* (DR3) | APO-3, DDR3, DR3, LARD, TNFRSF12, TR3, TRAMP, WSL-1, WSL-LR | TNF receptor superfamily member 25 | TL1A. Also associates with TRADD. |
| *EDA2R* (XEDAR) | EDA-A2R, EDAA2R, TNFRSF27, XEDAR | Ectodysplasin A2 receptor | EDA. Also associates with TRAF1, TRAF3, and TRAF6. |
| *EDAR* (EDAR) | DL, ED1R, ED3, ED5, EDA1R, EDA3 | Ectodysplasin A receptor | EDA. Also associates with TRAF1, TRAF2, and TRAF3. |
| *RELT* (RELT) | FLJ14993, TNFRSF19L | RELT TNF receptor | No TNF ligand. Associates with TRAF1. |
