# Supplementary material for: Targeting TNF/TNFR superfamilies in immune-mediated inflammatory diseases
Source: J Exp Med. 2024 Sep 19;221(11):e20240806. doi: 10.1084/jem.20240806 (PMC11413425; doi:10.1084/jem.20240806)
Supplement: Table S2 — shows select phase 3 clinical trials of FDA approved TNF-α antagonists and their results. [file JEM_20240806_TableS2.docx]

**Table S2.** **Select phase 3 clinical trials of FDA approved TNF-α antagonists and their results.**

| Drug (Trade name) | FDA approved indication (initial approval) | Representative phase 3 studies | Intervention | Primary endpoint(s) results |
| --- | --- | --- | --- | --- |
| Adalimumab (Humira) | Moderately to severely active CD in adults and pediatric patients 6 years of age and older (2007) | NCT00077779 (Colombel et al., 2007) | 854 patients with active CD received an induction dose of 80 mg subcutaneous adalimumab at week 0, and 40 mg adalimumab at week 2. Patients were then randomized at week 4 to receive adalimumab at 40 mg every week, or 40 mg every two weeks, or placebo. | **CDAI:** Higher rates of clinical remission in patients administered adalimumab 40 mg every two weeks (40%, *P*<0.001) and adalimumab 40 mg every week (47%, *P*<0.001) v. placebo (17%) at week 26. Higher rates of clinical remission in patients administered adalimumab 40 mg every two weeks (36%, *P*<0.001) and adalimumab 40 mg every week (41%, *P*<0.001) v. placebo (12%) at week 56. |
|  | Moderate-to-severe chronic plaque psoriasis who are candidates for systemic therapy or phototherapy, and when other systemic therapies are medically less appropriate (2008) | NCT00235820 (Saurat et al., 2008) | 271 patients with moderate-to-severe psoriasis received subcutaneous adalimumab at 40 mg every two weeks post a loading dose of 80 mg, 7.5 mg oral MTX, or placebo. | **PASI75:** Higher rates of disease improvement in patients administered adalimumab (79.6%) v. MTX (35.5%, *P*<0.001) and v. placebo (18.9%, *P*<0.001) at week 16. |
|  |  | NCT00237887 (Menter et al., 2008) | 1212 patients with moderate-to-severe plaque psoriasis received subcutaneous adalimumab at 40 mg every two weeks post a loading dose of 80 mg, or placebo. | **PASI75:** Higher rates of disease improvement in patients administered adalimumab (71%, *P*<0.001) v. placebo (7%) at week 16. |
|  | Adults with moderately to severely active RA (2002) | NCT00195702 (Keystone et al., 2004) | 619 patients with active RA who had an inadequate response to MTX received 40 mg adalimumab subcutaneously every two weeks, 20 mg adalimumab every week, or placebo, in addition to MTX. | **ACR20:** Better responses in patients administered adalimumab 40 mg every two weeks (63%, *P*<0.001) and 20 mg weekly (61%, *P*<0.001) v. placebo (30%) at week 24. **mTSS:** Lower change from baseline in mTSS in patients receiving adalimumab 40 mg every two weeks (0.1 ± 4.8, *P*<0.001) and 20 mg weekly (0.8 ± 4.9, *P*<0.001) v. placebo (2.7 ± 6.8) at week 52. **HAQ:** Greater improvement in patients administered adalimumab 40 mg every two weeks (-0.59, *P*<0.001) and 20 mg weekly (-0.61, *P*<0.001) v. placebo (-0.25) at week 52. |
|  |  | NCT00195663 (Breedveld et al., 2006) | 799 patients with active RA who never had been treated with MTX received 40 mg adalimumab subcutaneously every two weeks in addition to oral MTX, only weekly oral MTX, or only 40 mg adalimumab every two weeks. | **ACR50:** Better responses in patients with combination of adalimumab and MTX (62%) v. solo MTX (46%, *P*<0.001) and v. solo adalimumab (41%, *P*<0.001) at week 52. **mTSS:** Lower change from baseline in mTSS in patients receiving adalimumab and MTX (1.3) v. solo MTX (5.7, *P*<0.001) and v. solo adalimumab (3, *P*<0.001) at week 52. |
|  | Moderately to severely active UC in adults and pediatric patients 5 years of age and older (2005) | NCT00408629 | 518 patients with moderate-to-severe UC received 160 mg adalimumab subcutaneously at week 0, 80 mg at week 2, and 40 mg every two weeks, or placebo. | **Mayo score:** Higher rates of clinical remission in patients administered adalimumab (16.5%, *P*=0.019) v. placebo (9.3%) at week 8. Higher rates of clinical remission in patients administered adalimumab (17.3%, *P*=0.004) v. placebo (8.5%) at week 52. |
|  |  | NCT00385736 (Reinisch et al., 2011) | 576 patients with moderate-to-severe UC who had an inadequate response to corticosteroids and/or immunosuppressant received subcutaneous adalimumab - either 160 mg at week 0, 80 mg at week 2, and 40 mg at weeks 4 and 6, or 80 mg at week 0, and 40 mg at weeks 2, 4, and 6 - or placebo. | **Mayo score:** Higher rates of clinical remission in patients administered 160 mg adalimumab at week 0, 80 mg at week 2, and 40 mg at weeks 4 and 6 (18.5%, *P*=0.031) v. placebo (9.2%) at week 8. However, clinical remission was not significantly different in the group administered 80 mg adalimumab at week 0, and 40 mg at weeks 2, 4, and 6 (10.0%, *P*=0.833) v. placebo. |
| Certolizumab pegol (Cimzia) | Adults with moderately to severely active CD who have had an inadequate response to conventional therapy (2008) | NCT00152490 (Sandborn et al., 2007) | 662 adults with moderate-to-severe CD received 400 mg of certolizumab subcutaneously at weeks 0, 2, and 4, and then every 4 weeks, or placebo. | **CDAI:** Higher rates of clinical response in patients administered certolizumab (37%, *P*=0.04) v. placebo (26%) at week 6, within the patient subpopulation that had a baseline serum CRP level of at least 10 mg/L. Higher rates of clinical response in patients administered certolizumab (22%, *P*=0.05) v. placebo (12%) at both weeks 6 and 26, within the patient subpopulation that had a baseline serum CRP level of at least 10 mg/L. |
|  | Adults with moderate-to-severe plaque psoriasis who are candidates for systemic therapy or phototherapy (2018) | NCT02346240 (Lebwohl et al., 2018) | 559 patients with moderate-to-severe chronic psoriasis received subcutaneous certolizumab at 400 mg or 200 mg every two weeks for 16 weeks, subcutaneous etanercept at 50 mg twice weekly for 12 weeks, or placebo. | **PASI75:** Higher rates of disease improvement in patients administered certolizumab at 200 mg every two weeks (61.3%, *P*<0.0001) and 400 mg every two weeks (66.7%, *P*<0.0001) v. placebo (5.0%) at week 12. Etanercept treatment resulted in disease improvement in 53.3% of subjects. |
|  | Adults with moderately to severely active RA (2009) | NCT00160602 (Smolen et al., 2009) | 619 patients with active RA who had an incomplete response to MTX received 400 mg certolizumab subcutaneously at weeks 0, 2, and 4, followed by either 200 mg or 400 mg drug every two weeks for 24 weeks, or placebo, in addition to MTX. | **ACR20:** Better responses in patients administered certolizumab at 200 mg (57.3%, *P*<0.001) and 400 mg (57.6%, *P*<0.001) v. placebo (8.7%) at week 24. |
| Etanercept (Enbrel) | Chronic moderate to severe plaque psoriasis in patients 4 years or older (2004 - adult, 2016 - pediatric) | 20021642 (Papp et al., 2005) | 583 patients with moderate-to-severe psoriasis received 50 mg or 25 mg twice weekly of subcutaneous etanercept, or placebo. | **PASI75:** Higher rates of disease improvement in patients administered etanercept at 50 mg twice weekly (49%, *P*<0.0001), and at 25 mg twice weekly (34%, *P*<0.0001) v. placebo (3%) at week 12. |
|  | Moderately to severely active RA (1998) | NCT00393471 (Klareskog et al., 2004) | 686 patients with active RA who had an inadequate response to DMARDs other than MTX received 25 mg of subcutaneous etanercept, up to 20 mg/week oral MTX, or both. | **ACR-N:** Greater responses in patients administered etanercept and MTX combination (AUC of 18.3%-years) v. both etanercept alone (AUC of 14.7%-years, *P*<0.0001) and MTX alone (AUC of 12.2%-years, *P*<0.0001) at week 24. **mTSS:** Lower change from baseline in mTSS in patients receiving etanercept and MTX combination (-0.54) v. etanercept alone (0.52, *P*=0.0006) and MTX alone (2.80, *P*<0.0001) at week 52. |
| Golimumab (Simponi) | Moderate to severe UC with an inadequate response or intolerant to prior treatment or requiring continuous steroid therapy (2013) | NCT00487539 (Sandborn et al., 2014) | 1064 patients with moderately to severely active UC who had an inadequate response to conventional therapies received 200 mg and 100 mg or 400 mg and 200 mg of subcutaneous golimumab at weeks 0 and 2, or placebo. | **Mayo score:** Higher rates of clinical response in patients administered 200 mg/100 mg golimumab (51.0%, *P*<0.0001) and 400 mg/200 mg golimumab (54.9%, *P*<0.0001) v. placebo (30.3%) at week 6. |
|  | Adults with moderately to severely active RA, in combination with MTX (2009) | NCT00264550 (Keystone et al., 2009) | 444 patients with active RA despite treatment with MTX received MTX only, 100 mg subcutaneous golimumab only, or 50 mg and 100 mg of golimumab in combination with MTX. | **ACR20:** Better responses in patients administered 50 mg golimumab plus MTX (55.1%, *P*=0.001) and 100 mg golimumab plus MTX (56.2%, *P*<0.001) v. placebo plus MTX group (33.1%) at week 14. The corresponding response in 100 mg golimumab plus placebo group was 44.4%. **HAQ:** Better improvements from baseline in patients administered 50 mg golimumab plus MTX (0.38, *P*=0.001) and 100 mg golimumab plus MTX (0.50, *P*<0.001) v. placebo plus MTX group (0.13) at week 24. The corresponding response in 100 mg golimumab plus placebo group was 0.13. |
| Infliximab (Remicade) | Adults and pediatric patients with moderately to severely active CD who have had an inadequate response to conventional therapy (1998 - adult, 2006 - pediatric) | NCT00207662 (Hanauer et al., 2002) | 573 patients with moderate-to-severe active CD received a 5 mg/kg intravenous dose of infliximab at week 0, randomized at week 2 to receive either of placebo at weeks 2 and 6 and then every 8 weeks thereafter until week 46, repeat infusions of 5 mg/kg infliximab at the same timepoints, or 5 mg/kg infliximab at weeks 2 and 6 followed by 10 mg/kg. | **CDAI:** Higher rates of clinical remission in patients administered infliximab (39% and 45%, both *P*<0.003) v. placebo (21%) at week 30. Median time to loss of response up to week 54 was higher in infliximab treatment groups (38 weeks and >54 weeks, both *P*<0.002) v. placebo (19 weeks). |
|  | Adults with chronic plaque psoriasis who are candidates for systemic therapy and when other systemic therapies are medically less appropriate (2006) | NCT00106834 (Reich et al., 2005) | 378 patients with moderate-to-severe psoriasis received 5 mg/kg intravenous infliximab, or placebo at weeks 0, 2, and 6, and then every 8 weeks up to week 46. At week 24, the placebo group crossed over to drug treatment. | **PASI75:** Higher rates of disease improvement in patients administered infliximab (80%, *P*<0.0001) v. placebo (3%) at week 10. |
|  | Adults with moderately to severely active RA, in combination with MTX (1999) | NCT00236028 (St Clair et al., 2004) | 1049 patients with active RA and no prior treatment with MTX or a TNF-α inhibitor received 3 mg/kg or 6 mg/kg intravenous infliximab at weeks 0, 2, and 6, and every 8 weeks thereafter through week 46, or placebo, concomitant with MTX (7.5mg/week, increased to 20mg/week by week 8). | **ACR-N:** Median percentage higher in patients administered 3 mg/kg (38.9%, *P*<0.001) and 6 mg/kg (46.7%, *P*<0.001) infliximab v. placebo (26.4%) at week 54. **mTSS:** Lower change from baseline in mTSS in patients receiving 3 mg/kg (0.4 ± 5.8, *P*<0.001) and 6 mg/kg (0.5 ± 5.6, *P*<0.001) infliximab v. placebo (3.7 ± 9.6) at week 54. |
|  |  | NCT00269867 (Lipsky et al., 2000) | 428 patients with active RA despite MTX therapy received intravenous infliximab at 3 mg/kg or 10 mg/kg every 4 or 8 weeks through week 54, or placebo, concomitant with oral MTX. | **ACR20:** Better responses in patients administered all tested doses of infliximab, with the best improvement in both 10 mg/kg doses (59%, *P*<0.001) v. placebo (17%) at week 54. **mTSS:** Lower change from baseline in mTSS in patients receiving any of the tested doses of infliximab, with the best improvement in the 10 mg/kg dose administered every 4 weeks eliciting a change of -0.7 ± 3.8 (*P*<0.001) v. placebo (7.0 ± 10.3) at week 54. |
|  | Adults and pediatric patients with moderately to severely active UC who have had an inadequate response to conventional therapy (2005 - adult, 2011 - pediatric) | NCT00036439 (Rutgeerts et al., 2005) | 364 patients with moderate-to-severe active UC despite treatment with concurrent medications received infliximab at 5 mg/kg or 10 mg/kg intravenous at weeks 0, 2, and 6, and then every eight weeks through week 46, or placebo. | **Mayo score:** Higher rates of clinical response in patients administered 5 mg/kg (69%, *P*<0.001), or 10 mg/kg (61%, *P*<0.001) infliximab v. placebo (37%) at week 8. |
|  |  | NCT00096655 (Rutgeerts et al., 2005) | 364 patients with moderate-to-severe active UC despite treatment with concurrent medications received infliximab at 5 mg/kg or 10 mg/kg intravenous at weeks 0, 2, and 6, and then every eight weeks through week 22, or placebo. | **Mayo score:** Higher rates of clinical response in patients administered 5 mg/kg (64%, *P*<0.001), or 10 mg/kg (69%, *P*<0.001) infliximab v. placebo (29%) at week 8. |

ACR, American College of Rheumatology; AUC, Area Under the Curve; CD, Crohn’s Disease; CDAI, Crohn’s Disease Activity Index; HAQ, Health Assessment Questionnaire; mTSS, modified Total Sharp x-ray Score; MTX, Methotrexate; PASI, Psoriasis Area and Severity Index; RA, Rheumatoid Arthritis; UC, Ulcerative Colitis.

**References**

Breedveld, F.C., M.H. Weisman, A.F. Kavanaugh, S.B. Cohen, K. Pavelka, R. van Vollenhoven, J. Sharp, J.L. Perez, and G.T. Spencer-Green. 2006. The PREMIER study: A multicenter, randomized, double-blind clinical trial of combination therapy with adalimumab plus methotrexate versus methotrexate alone or adalimumab alone in patients with early, aggressive rheumatoid arthritis who had not had previous methotrexate treatment. *Arthritis Rheum* 54:26-37.

Colombel, J.F., W.J. Sandborn, P. Rutgeerts, R. Enns, S.B. Hanauer, R. Panaccione, S. Schreiber, D. Byczkowski, J. Li, J.D. Kent, and P.F. Pollack. 2007. Adalimumab for maintenance of clinical response and remission in patients with Crohn's disease: the CHARM trial. *Gastroenterology* 132:52-65.

Hanauer, S.B., B.G. Feagan, G.R. Lichtenstein, L.F. Mayer, S. Schreiber, J.F. Colombel, D. Rachmilewitz, D.C. Wolf, A. Olson, W. Bao, and P. Rutgeerts. 2002. Maintenance infliximab for Crohn's disease: the ACCENT I randomised trial. *Lancet* 359:1541-1549.

Keystone, E.C., M.C. Genovese, L. Klareskog, E.C. Hsia, S.T. Hall, P.C. Miranda, J. Pazdur, S.-C. Bae, W. Palmer, J. Zrubek, M. Wiekowski, S. Visvanathan, Z. Wu, and M.U. Rahman. 2009. Golimumab, a human antibody to tumour necrosis factor α given by monthly subcutaneous injections, in active rheumatoid arthritis despite methotrexate therapy: the GO-FORWARD Study. *Annals of the Rheumatic Diseases* 68:789-796.

Keystone, E.C., A.F. Kavanaugh, J.T. Sharp, H. Tannenbaum, Y. Hua, L.S. Teoh, S.A. Fischkoff, and E.K. Chartash. 2004. Radiographic, clinical, and functional outcomes of treatment with adalimumab (a human anti–tumor necrosis factor monoclonal antibody) in patients with active rheumatoid arthritis receiving concomitant methotrexate therapy: A randomized, placebo-controlled, 52-week trial. *Arthritis & Rheumatism* 50:1400-1411.

Klareskog, L., D. van der Heijde, J.P. de Jager, A. Gough, J. Kalden, M. Malaise, E.M. Mola, K. Pavelka, J. Sany, L. Settas, J. Wajdula, R. Pedersen, S. Fatenejad, and M. Sanda. 2004. Therapeutic effect of the combination of etanercept and methotrexate compared with each treatment alone in patients with rheumatoid arthritis: double-blind randomised controlled trial. *The Lancet* 363:675-681.

Lebwohl, M., A. Blauvelt, C. Paul, H. Sofen, J. Węgłowska, V. Piguet, D. Burge, R. Rolleri, J. Drew, L. Peterson, and M. Augustin. 2018. Certolizumab pegol for the treatment of chronic plaque psoriasis: Results through 48 weeks of a phase 3, multicenter, randomized, double-blind, etanercept- and placebo-controlled study (CIMPACT). *J Am Acad Dermatol* 79:266-276.e265.

Lipsky, P.E., D.M. van der Heijde, E.W. St Clair, D.E. Furst, F.C. Breedveld, J.R. Kalden, J.S. Smolen, M. Weisman, P. Emery, M. Feldmann, G.R. Harriman, and R.N. Maini. 2000. Infliximab and methotrexate in the treatment of rheumatoid arthritis. Anti-Tumor Necrosis Factor Trial in Rheumatoid Arthritis with Concomitant Therapy Study Group. *N Engl J Med* 343:1594-1602.

Menter, A., S.K. Tyring, K. Gordon, A.B. Kimball, C.L. Leonardi, R.G. Langley, B.E. Strober, M. Kaul, Y. Gu, M. Okun, and K. Papp. 2008. Adalimumab therapy for moderate to severe psoriasis: A randomized, controlled phase III trial. *J Am Acad Dermatol* 58:106-115.

Papp, K.A., S. Tyring, M. Lahfa, J. Prinz, C.E. Griffiths, A.M. Nakanishi, R. Zitnik, P.C. van de Kerkhof, L. Melvin, and G. Etanercept Psoriasis Study. 2005. A global phase III randomized controlled trial of etanercept in psoriasis: safety, efficacy, and effect of dose reduction. *Br J Dermatol* 152:1304-1312.

Reich, K., F.O. Nestle, K. Papp, J.P. Ortonne, R. Evans, C. Guzzo, S. Li, L.T. Dooley, C.E. Griffiths, and E.s. investigators. 2005. Infliximab induction and maintenance therapy for moderate-to-severe psoriasis: a phase III, multicentre, double-blind trial. *Lancet* 366:1367-1374.

Reinisch, W., W.J. Sandborn, D.W. Hommes, G. D'Haens, S. Hanauer, S. Schreiber, R. Panaccione, R.N. Fedorak, M.B. Tighe, B. Huang, W. Kampman, A. Lazar, and R. Thakkar. 2011. Adalimumab for induction of clinical remission in moderately to severely active ulcerative colitis: results of a randomised controlled trial. *Gut* 60:780-787.

Rutgeerts, P., W.J. Sandborn, B.G. Feagan, W. Reinisch, A. Olson, J. Johanns, S. Travers, D. Rachmilewitz, S.B. Hanauer, G.R. Lichtenstein, W.J. de Villiers, D. Present, B.E. Sands, and J.F. Colombel. 2005. Infliximab for induction and maintenance therapy for ulcerative colitis. *N Engl J Med* 353:2462-2476.

Sandborn, W.J., B.G. Feagan, C. Marano, H. Zhang, R. Strauss, J. Johanns, O.J. Adedokun, C. Guzzo, J.F. Colombel, W. Reinisch, P.R. Gibson, J. Collins, G. Järnerot, T. Hibi, and P. Rutgeerts. 2014. Subcutaneous golimumab induces clinical response and remission in patients with moderate-to-severe ulcerative colitis. *Gastroenterology* 146:85-95; quiz e14-85.

Sandborn, W.J., B.G. Feagan, S. Stoinov, P.J. Honiball, P. Rutgeerts, D. Mason, R. Bloomfield, and S. Schreiber. 2007. Certolizumab pegol for the treatment of Crohn's disease. *N Engl J Med* 357:228-238.

Saurat, J.H., G. Stingl, L. Dubertret, K. Papp, R.G. Langley, J.P. Ortonne, K. Unnebrink, M. Kaul, A. Camez, and f.t.C.S. Investigators. 2008. Efficacy and safety results from the randomized controlled comparative study of adalimumab vs. methotrexate vs. placebo in patients with psoriasis (CHAMPION). *British Journal of Dermatology* 158:558-566.

Smolen, J., R.B. Landewé, P. Mease, J. Brzezicki, D. Mason, K. Luijtens, R.F. van Vollenhoven, A. Kavanaugh, M. Schiff, G.R. Burmester, V. Strand, J. Vencovsky, and D. van der Heijde. 2009. Efficacy and safety of certolizumab pegol plus methotrexate in active rheumatoid arthritis: the RAPID 2 study. A randomised controlled trial. *Ann Rheum Dis* 68:797-804.

St Clair, E.W., D.M. van der Heijde, J.S. Smolen, R.N. Maini, J.M. Bathon, P. Emery, E. Keystone, M. Schiff, J.R. Kalden, B. Wang, K. Dewoody, R. Weiss, and D. Baker. 2004. Combination of infliximab and methotrexate therapy for early rheumatoid arthritis: a randomized, controlled trial. *Arthritis Rheum* 50:3432-3443.
